# CSF1R regulates schizophrenia-related stress response and vascular association of microglia/macrophages

**DOI:** 10.1186/s12916-023-02959-8

**Published:** 2023-08-04

**Authors:** Ling Yan, Yanli Li, Fengmei Fan, Mengzhuang Gou, Fangling Xuan, Wei Feng, Keerthana Chithanathan, Wei Li, Junchao Huang, Hongna Li, Wenjin Chen, Baopeng Tian, Zhiren Wang, Shuping Tan, Alexander Zharkovsky, L. Elliot Hong, Yunlong Tan, Li Tian

**Affiliations:** 1 Institute of Biomedicine and Translational Medicine, Faculty of Medicine, University of Tartu, Tartu, Estonia; 2 Psychiatry Research Centre, Beijing Huilongguan Hospital, Peking University Health Science Center, Peking University HuiLongGuan Clinical Medical School, Beijing, P. R. China; 3 Department of Psychiatry, School of Medicine, Maryland Psychiatric Research Center, University of Maryland, Baltimore, USA

**Keywords:** Microglia/Brain macrophages, CSF1R, Angiogenesis, First episode schizophrenia, Stress, Anxiety

## Abstract

**Background:**

Microglia are known to regulate stress and anxiety in both humans and animal models. Psychosocial stress is the most common risk factor for the development of schizophrenia. However, how microglia/brain macrophages contribute to schizophrenia is not well established. We hypothesized that effector molecules expressed in microglia/macrophages were involved in schizophrenia via regulating stress susceptibility.

**Methods:**

We recruited a cohort of first episode schizophrenia (FES) patients (*n* = 51) and age- and sex-paired healthy controls (HCs) (*n* = 46) with evaluated stress perception. We performed blood RNA-sequencing (RNA-seq) and brain magnetic resonance imaging, and measured plasma level of colony stimulating factor 1 receptor (CSF1R). Furthermore, we studied a mouse model of chronic unpredictable stress (CUS) combined with a CSF1R inhibitor (CSF1Ri) (*n* = 9 ~ 10/group) on anxiety behaviours and microglial biology.

**Results:**

FES patients showed higher scores of perceived stress scale (PSS, *p* < 0.05), lower blood CSF1R mRNA (FDR = 0.003) and protein (*p* < 0.05) levels, and smaller volumes of the superior frontal gyrus and parahippocampal gyrus (both FDR < 0.05) than HCs. In blood RNA-seq, *CSF1R*-associated differentially expressed blood genes were related to brain development. Importantly, *CSF1R* facilitated a negative association of the superior frontal gyrus with PSS (*p* < 0.01) in HCs but not FES patients. In mouse CUS+CSF1Ri model, similarly as CUS, CSF1Ri enhanced anxiety (both *p* < 0.001). Genes for brain angiogenesis and intensity of CD31^+^-blood vessels were dampened after CUS-CSF1Ri treatment. Furthermore, CSF1Ri preferentially diminished juxta-vascular microglia/macrophages and induced microglia/macrophages morphological changes (all *p* < 0.05).

**Conclusion:**

Microglial/macrophagic CSF1R regulated schizophrenia-associated stress and brain angiogenesis.

**Supplementary Information:**

The online version contains supplementary material available at 10.1186/s12916-023-02959-8.

## Background

Schizophrenia is a complex neurodevelopmental disorder usually caused by environmental insults on genetically predisposed individuals [1], which can be recapitulated in animal models [2]. Psychosocial stressors have been shown to trigger or exacerbate symptoms of schizophrenia [3, 4], and heightened stress response usually precedes the onset of psychosis in both schizophrenia patients [5, 6] and rodents [7].

Dystrophies of the cortical and associated limbic structures are frequently observed in both schizophrenia patients [8–10] and animal models of chronic psychosocial stress [11]. Neurobiological substrates underlying stress-induced brain changes may include both impaired neuronal projections across different brain structures [12] and enhanced local microglia/astrocytes-mediated neuroinflammation [13, 14].

Glia are important regulators for brain structural and functional connectivity. Besides, borderline/barrier-associated macrophages also constitute an important cellular sentinel in the normal adult brain [15]. Their overactivation can enhance synaptic pruning, prevent angiogenesis and neurogenesis, and induce neuronal and myelinic loss [16–18]. Nevertheless, immune cells, especially microglia, are also beneficial for brain homeostasis with neuro-protective functions and may contribute to stress adaptation, as demonstrated by others and us in mice [19, 20].

Colony stimulating factor 1 receptor (CSF1R) is a receptor tyrosine kinase crucial for development and functions of myeloid cells including microglia and monocytes [21]. Both human subjects with *CSF1R* loss-of-function mutation and *Csf1r*^−/−^ mice display shortened lifespan, loss of microglia and macrophages, and neurodevelopmental abnormalities [22, 23]. While genetic or pharmacological inhibition of CSF1R (CSF1Ri) produced no gross behavioural changes in adult animals [24], a recent study demonstrated that *Csf1r* haplo-deficiency was anxiogenic to mice [25]. CSF1Ri-induced microglial ablation enhanced fear learning and memory [26, 27] while microglial repopulation corrected repetitive behaviour and social deficits [28, 29]. Furthermore, CSF1 ameliorated depressive-like behaviour in mice after chronic unpredictable stress (CUS) [30]. Lower CSF1R in the post-mortem brains of chronic schizophrenia patients was reported [31–33]. However, the exact role of CSF1R in schizophrenia in association with psychosocial stress has remained unclear.

We earlier reported that cumulative stress was associated with cortical thinning and cognitive deficits in first episode schizophrenia (FES) patients [34]. In the present study, we hypothesized that CSF1R-involved microglial/macrophagic functions were associated with schizophrenia-related stress modulation via regulating the brain cortical and subcortical structures. To better understand the role of microglia or myeloid cells in schizophrenia-related stress regulation, we first studied a cohort of FES patients showing higher stress perception compared to healthy controls (HCs) by measuring their blood transcriptomics, plasma CSF1R levels, and brain structures. We further used a CUS mouse model combined with pharmacological CSF1Ri to characterize the response of microglia/macrophages by brain RNA-seq, immunohistochemistry, flow cytometry, and animal anxiety tests.

## Methods

### Clinical demographics and measures

FES Patients (*n* = 128) recruited for this study were from the Beijing Hui Long Guan Hospital. Patients were diagnosed schizophrenia according to the Structured Clinical Interview for DSM-IV (SCID) independently by two psychiatrists. Inclusion criteria were: (1) 18–54-year-old Han Chinese; (2) illness duration ≤ 3 years (< 1 year on average); and (3) un-medicated or < 2 weeks of anti-psychotic medication at the time of blood draw. Age- and sex-matched HCs (*n* = 111) were recruited from local community. Candidates who unmet recruitment criteria were excluded. Additional exclusion criteria included: (1) other psychiatric disorders diagnosed according to the DSM-IV Axis I; (2) severe physical illness; (3) recent infection or treatment with physiotherapy or psychotherapy; (4) mental retardation or serious nervous system disease; and (5) lactation or pregnancy. All participants provided written informed consent. The study was approved by the Institutional Ethical Committee of Beijing Huilongguan Hospital with license No. 2017–49. Participants’ (FES: *n* = 51, HC: *n* = 46) past traumatic experiences were evaluated by Childhood Trauma Questionnaire (CTQ), a 29-item self-reported questionnaire of a retrospective measure encompassing five adverse factors [35], validated in Chinese [36]. Participants’ stress levels were evaluated based on perceived stress scale (PSS), a 14-item self-reported questionnaire measuring feelings and thoughts during the last month [37], validated in Chinese [38]. Positive and Negative Syndrome Scale total scores (PANSSt) were measured independently by two psychiatrists. For details, see supplementary material.

### Magnetic resonance imaging (MRI) acquisition and processing

Brain structural MRI data were acquired using a Siemens Prisma 3.0 T MRI scanner with a 64-channel head coil. Foam pads were used to minimize head motions. Sagittal three-dimensional magnetization-prepared rapid acquisition gradient echo (MPRAGE) was used to collect each participant’s anatomical data following the ENIGMA protocol with FreeSurfer software [39, 40]: repetition time (TR)/echo time (TE)/inversion time (TI) = 2530/2.98/1100 ms, flip angle (FA) = 7º, field of view (FOV) = 256 × 224 mm^2^, Pixel/gap size = 1/0 mm, matrix size = 256 × 224 bit. After scanning, two radiologists evaluated image quality and if there were significant artefacts, images were recollected. Intracranial volume (ICV) and regional volumes of bi-hemispheric cerebral cortical/subcortical structures were measured.

### Human and mouse RNA sequencing (RNA-seq) and real time quantitative PCR (RT-QPCR)

Human blood (5 ml) was collected between 7–9 am after overnight fasting using PAXgene™ blood RNA tubes (Applied Biosystems). Tubes were shaken vigorously for at least 10 s after sampling and immediately stored at -80 °C. Total RNAs from human blood (Applied Biosystems) and mouse PFC (Molecular Research Center) were extracted, quantified, and assessed for purity using NanoDrop spectrophotometry (ThermoFisher), and immediately sent to the Beijing Genomics institution (BGI) for messenger RNA-seq (after globin mRNA removal and quality control) on the BGIseq-500 platform. RNA-seq data of at least 20 M clean reads were analyzed in the Galaxy and NetworkAnalyst platforms [41] using DESEQ2. Data with variance percentile rank < 15% and counts < 4 were filtered out. Log2 fold changes (Log2FC) for differentially expressed genes (DEGs) with Benjamini-Hochberg’s false discovery rate (FDR) < 0.05 were analyzed for gene ontology biological pathway (GO-BP) in DAVID (https://david.ncifcrf.gov/) and protein–protein interaction (PPI) in STRING (https://string-db.org/cgi/input.pl). Genesets (GS393224, GS393415, and GS393709) retrieved from GeneWeaver (https://geneweaver.org/) and annotated for CSF1R-mediated association with human brain development were further explored for overlapping blood RNA-seq DEGs. Total RNAs were reversely transcribed (Thermo Scientific) and RT-QPCR was performed with corresponding primers (Additional file 1: Table S1) and qPCR Supermix (Solis BioDyne) on a QPCR instrument (Applied Biosystems). Normalized target-*actin* ΔCt values were further quantified as exponential fold-changes against averaged ΔCt of Ctr group (2^-ΔΔCt).

### Plasma CSF1R protein detection

Blood samples (5 ml) were collected as above described into EDTA-K2 disposable vacuum collection tubes (Beijing Dongfang Jianfeng Technology Co. Ltd.). Plasma samples were separated by centrifugation at 4000 rpm for 10 min, which were immediately stored at -80 °C until assayed. CSF1R protein was measured by sandwich enzyme-linked immunosorbent assay (ELISA) kit (#RX-XQ-EN13238, Beijing Rongxin Zhihe Biotechnology Co. Ltd.). Each sample (FES: *n* = 126, HC: *n* = 102) was measured in duplicates. The intra-plate and inter-plate variation coefficients for the ELISA were 10% and 15%, respectively.

### Mouse CUS and CSF1Ri (PLX3397) treatment procedures

Wild-type C57BL/6NTac male mice (3-month-old, Taconic) were bred under standard breeding conditions in laboratory animal facility at the Institute of Biomedicine and Translational Medicine, University of Tartu with the license No. 171. After a week (wk) of transfer adaptation, mice were randomly assigned into 4 groups (*n* = 9 ~ 10/group): Control (Ctr)-Vehicle (Veh), CUS-Veh, Ctr-CSF1Ri, and CUS-CSF1Ri, and subject to CUS/Ctr and CSF1Ri (PLX3397)/Veh treatments. CUS was established by daily applied one of seven random stressors for 8 wk, as we recently described [42]. PLX3397 (HY-16749/CS-4256, MedChemExpress) was dissolved in DMSO (D8418, Sigma-Aldrich) and freshly diluted with corn oil (#8267, Sigma-Aldrich). Drug-treated mice were daily fed with Veh or PLX3397 (~ 120 mg/kg bodyweight) in Nutella, as reported [43, 44], for 2 wk starting from the 7^th^ wk of CUS. Behavioural experiments were performed at the 8^th^ wk (see Fig. 3A).

### Open field test (OFT)

Mice were habituated to ~ 250 lux room light for 1 hour (h). Individual mouse was measured for distance and time travelled in different zones of a digital box (44.8 × 44.8 × 45 cm) via a software (Technical & Scientific Equipment GmbH) for 30 minutes (min). The floor of the box was cleaned with 70% ethanol and dried thoroughly after each mouse.

### Elevated plus maze (EPM)

EPM consisted of open and closed arms (30 × 5 cm each) intersected at a central 5 × 5 cm square platform elevated to a height of 80 cm. Mice were habituated to ~ 40 lux room light for 1 h. Individual mouse was placed on the central platform facing the open arm and recorded for time spent on open/close arms by a software (EthoVision XT, Noduls) for 5 min. The arms were cleaned with 70% ethanol and dried thoroughly after each mouse.

### Brain tissue processing and immunohistochemistry

Coronal cryosections containing the prefrontal cortex (PFC) and hippocampus (HPC) in 40 μm-thickness were incubated with primary antibodies including rabbit anti-IBA1 (#SKL6615, Wako) and rat anti-CD31 (#553,370, BD Pharmingen) in PBS blocking buffer overnight at 4 °C, followed by goat anti-rabbit IgG H&L-AlexaFluor488 (#ab175471, Abcam) and goat anti-rat IgG H&L-AlexaFluor546 (#119,170, Jackson ImmunoResearch) for 2 h at room temperature and then in 0.1 μg/ml DAPI (#ACRO202710100, VWR) for 5 min, and finally mounted to glass slides with Fluoromount™ Aqueous Mounting Medium (# F4680-25ML, Sigma-Aldrich). Z-stack images were taken by a FV1200MPE laser scanning microscope at 60 × magnification (Olympus). After 3D image reconstruction, CD31^+^-area/whole image area*100% was calculated. IBA1^+^-microglia/macrophages whose cell soma located within the range of vascular radius surrounding a CD31^+^-blood vessel were defined as vessel-associated microglia/macrophages (VAMs) and others as nonvessel-associated microglia/macrophages (NVAMs). Fluorescent intensities of CD31^+^- and IBA1^+^-areas and microglial number (No.) and morphology were measured using ImageJ (*n* = 3-mice/12-sections/40 ~ 200-cells per group).

### Flow cytometry

Hippocampal homogenates were washed, centrifuged at 500 g for 5 min, and blocked with PBS + 10% rat serum for 1 h, then stained with flow markers (BioLegend and Miltenyi) of anti-mouse Csf1r-Brilliant Violet (BV)605 (135517, BioLegend), CD11b-BV421 (101251, BioLegend), CD45-BV650 (103151, BioLegend), Glast-APC (130–123-555, Miltenyi), and O4-PE (130–117-357, Miltenyi) for 1 h. Washed cells were resuspended in 500 µl PBS and acquired with a Fortessa flow cytometer (BD Bioscience). Data were analyzed by Kaluza v2.1 software (Beckman Coulter). Astrocytes were defined as Glast^+^ cells, oligodendrocyte precursor cells (OPCs) as O4^+^ cells, microglia as CD45^low^CD11b^hi^ cells. The % of glia among total brain cells and mean fluorescent intensity (MFI) of Csf1r per microglia were measured and total Csf1r protein level was calculated as MFI x microglial No.

### Statistical analysis

Data distributions were examined by Shapiro–Wilk’s test. For human data, ANOVA or Mann–Whitney U test was used for continuous variables and chi-squared test for categorical variables. ANCOVA with age and sex as covariates was conducted for plasma CSF1R and MRI data, with white blood cells and ICV included as additional covariates for them, respectively, and *p* values for multiple comparisons were corrected by FDR. Correlation was analyzed by Pearson’s or Spearman’s method. Relationships among CSF1R level, cortical size, and PSS score were evaluated using linear regression and moderator analysis by PROCESS-v3.5 in SPSS-v27.0 (IBM), controlled by age, sex, and ICV. For animal data, two-way ANOVA was used to examine the interaction between CUS and CSF1Ri, with Bonferroni's correction for post hoc comparisons. Figures were prepared in GraphPad Prism-v8.0.1 and online (http://www.bioinformatics.com.cn/). Data were presented as mean ± SEM and *p* or FDR < 0.05 was considered statistically significant.

## Results

### Blood CSF1R mRNA and protein levels were lowered in FES patients

**Fig. 1:**
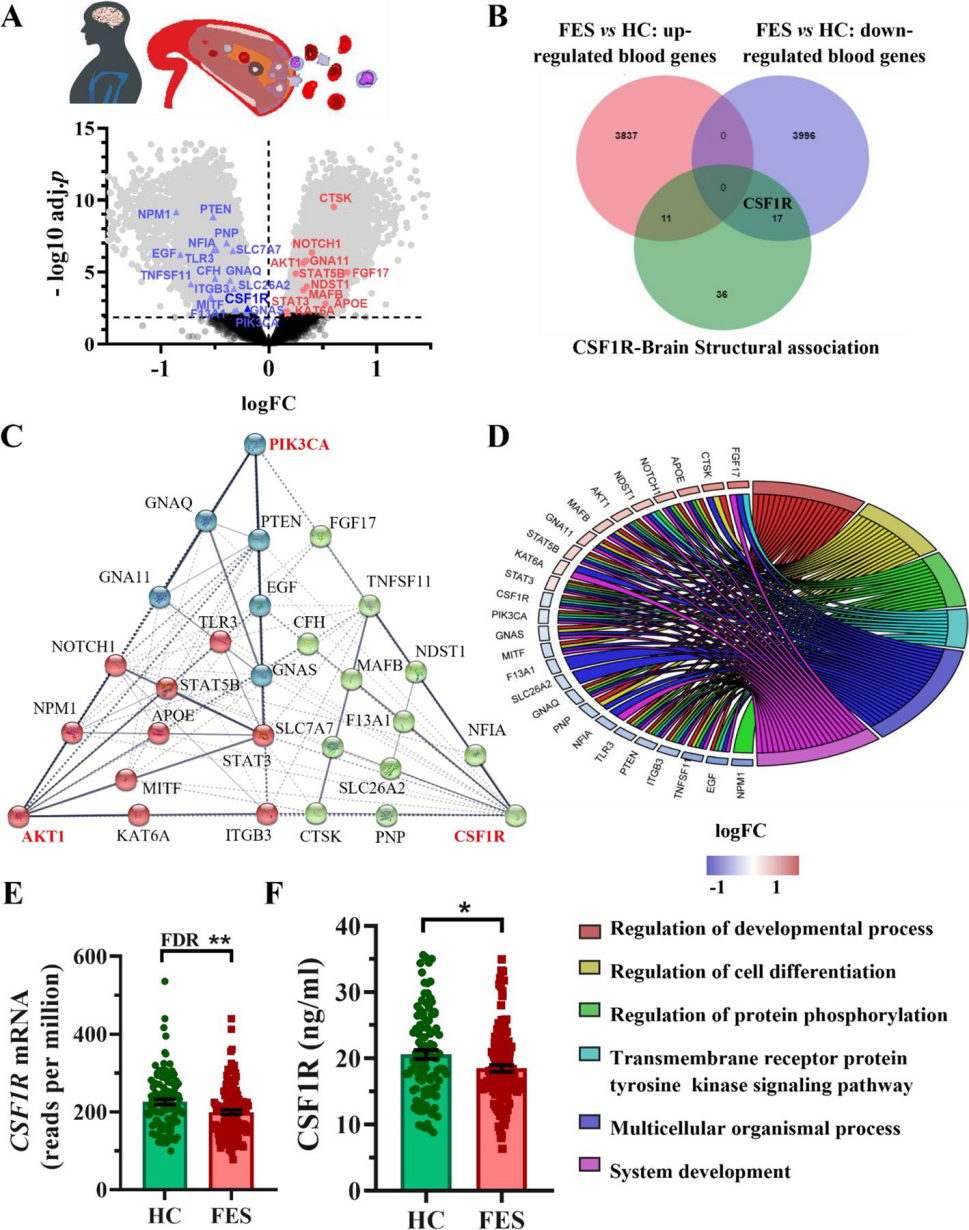
Blood CSF1R and DEGs related to brain development were decreased in FES patients. (**A**) A volcano plot highlights 28 blood DEGs in FES patients versus HCs. Genes with FDR < 0.01 are colored (*n* = 128 + 111). See also Additional file 1: Table S2. (**B**) Venn diagram illustrates the 28 DEGs that are annotated to be associated with human brain structural development in GeneWeaver. (**C**) PPI analysis shows functional interactions among the 28 DEGs, with confidence threshold = 0.4 and cluster k-means = 3. The 3 clusters are coloured differently, with the hub gene in each cluster highlighted in red color and bold at each triangular tip. Line between nodes features the type/strength of an interaction according to annotations in String v11. (**D**) A chord plot shows top 6 overrepresented GO-BP subontology for the 28 DEGs associated with brain development. Genes are ordered according to the observed Log2FC and linked to their assigned terms via coloured ribbons. See also Additional file 1: Table S3 for pathway analysis. (**E**) CSF1R mRNA level (*n* = 128 + 111). (**F**) CSF1R protein level (*n* = 126 + 102). Data presented as mean ± SEM; * *p* < 0.05 (ANCOVA), ** FDR < 0.01. CSF1R: colony stimulating factor 1 receptor; DEGs: differentially expressed genes; FES: First episode schizophrenia; HCs: healthy controls; PPI: protein–protein interaction

We earlier had collected whole blood samples from a cohort of 128 FES patients and 111 HCs, including the subjects of our current study, and identified 9062 DEGs by RNA-seq [45]. Hence, we first explored this dataset and noted downregulated *CSF1R* mRNA (Fig. 1A). As the CSF1R is known to be important for brain development, we thereby explored functional genomic data in GeneWeaver and retrieved 64 *CSF1R*-associted human brain developmental genes. To better depict which of the 64 candidate genes were changed in our patients’ blood, we overlapped them with blood RNA-seq DEGs and found 11 up-regulated and 17 down-regulated genes including the *CSF1R* (Fig. 1A & 1B; Additional file 1: Table S2). To depict functional relationships among the 28 DEGs, we studied their interactions and GO enrichment with PPI analysis, showing three different functional clusters, with the *PIK3CA*, *AKT1* and *CSF1R* as the hub genes, respectively (Fig. 1C), and the top-ranked GO-BP pathway being regulation of developmental process (Fig. 1D**; **Additional file 1: Table S3). To validate the *CSF1R* downregulation in the FES patients compared to the HCs as shown by  the RNA-seq (FDR = 0.003; Fig. 1E (*n* = 128 + 111)), we further measured the plasma CSF1R protein and confirmed it in both patient cohorts (*p* < 0.05; Fig. 1F (*n* = 126 + 102), Table 1 (*n* = 50 + 44)).

**Table 1:** Demographic characteristics of FES patients and HCs

**Demographics**	**FES** (*n* = 51)	**HC** (*n* = 46)	**F or χ2**	***P***
Sex (M/F)	18/33	23/23	2.143	0.143
Age (years)^a^	30.59 (1.18)	34.00 (1.46)	1388.000	0.120
Education (years)^a^	12.75 (0.49)	13.28 (0.33)	1298.500	0.354
CTQsum^a^	81.55 (5.49)	70.02 (4.78)	727.500	0.125
PSSsum^a^	23.14 (0.99)	21.39 (0.67)	893.000	**0.043**
Age of illness onset (years)	29.27 (1.17)			
Illness duration (months)	11.70 (2.09)			
PANSSt	75.69 (2.03)			
White blood cells (10^6^/ml)^a^	7.60 (1.08)	6.01 (0.25)	856.000	0.085
Plasma CSF1R (ng/ml)	18.55 (1.03)	21.26 (0.766)	5.539	**0.021**

All data were reported as mean (SEM), *FES* first episode schizophrenia, *HC* healthy control, *FDR* false discovery rate, *CSF1R* colony stimulating factor 1 receptor; *CTQ* childhood trauma questionnaire; *PSS* perceived stress scale; *PANSSt* positive and negative symptom scale total score. CSF1R (ANCOVA controlled by age, sex, and white blood cells); Significant *p* values are shown in bold texts. ^a^Mann-Whitney U test

### FES patients showed higher perceived stress and smaller cerebral cortical regions than HCs

**Fig. 2:**
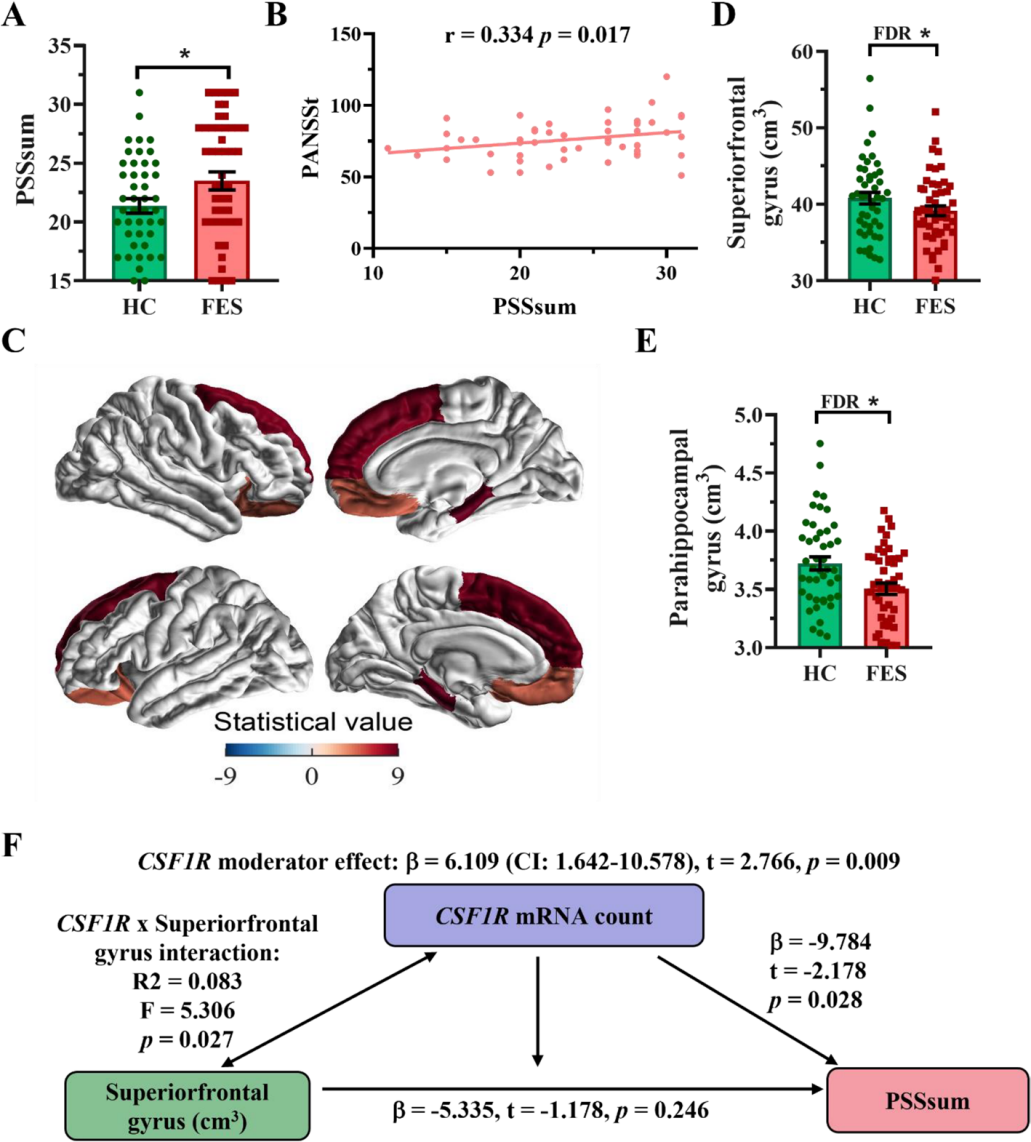
*CSF1R* facilitated a negative association of the superior frontal gyrus with PSSsum in HCs but not FES patients. (**A**) PSSsum (*n* = 51 + 46). (**B**) Correlation of PANSSt and PSSsum in FES patients (Spearman’s correlation). (**C**) Exemplary MRI images, color gradient is based on the statistical *F* values (detailed in Table 2) of group comparison. (**D**) Volume of the superior frontal gyrus and (**E**) Volume of the parahippocampal gyrus. (**F**) Blood *C*SF1R mRNA level fully moderated the negative association of the superior frontal gyral volume (independent variable) with the PSSsum score (dependent variable), controlled by age, sex, and ICV in HCs. Data presented as mean ± SEM; * FDR or p < 0.05 (ANCOVA). See also Figure S1. CSF1R: colony stimulating factor 1 receptor; DEGs: differentially expressed genes; FES: First episode schizophrenia; HCs: healthy controls; PSSsum: perceived stress scale summation score

We also studied those participants who were with both MRI and PSS evaluations. Participants’ demographic and clinical data are listed in Table 1 (*n* = 51 + 46). The FES patients and the HCs were not statistically different in age, sex, education years and CTQ score (all *p* > 0.05). However, compared with the HCs, the FES patients had lower CSF1R protein level (*p* < 0.05; Table 1) and higher PSSsum score (Fig. 2A, Table1), which was positively correlated with PANSSt score (r = 0.334, *p* < 0.05; Fig. 2B).

We next studied 8 key stress-related brain regions, including the PFC subareas, HPC, and HPC-associated entorhinal and parahippocampal gyri (Fig. 2C, Table 2), and observed reduced volumes in the superior frontal gyrus (*p* < 0.005, FDR = 0.02; Fig. 2D) and parahippocampal gyrus (*p* < 0.004, FDR = 0.032; Fig. 2E) in the FES patients compared to the HCs. Additionally, the orbital frontal gyrus also showed nominal significance in reduction in the FES patients compared to the HCs (*p* < 0.05; Table 2). The HPC and other cortical structures did not show significant differences, however (Table 2).

**Table 2:** Cerebral cortical volumes (cm^3^) in FES patients and HCs

**Cortical regions**	**FES** (n = 51)	**HC** (n = 46)	**F**	***P*-value**	**FDR**
Anteriocingulate gyrus	7.729 ± 0.126	7.927 ± 0.133	1.124	0.292	0.467
Entorhinal gyrus	3.152 ± 0.069	3.136 ± 0.069	0.442	0.508	0.581
Hippocampus	8.039 ± 0.084	8.109 ± 0.096	0.156	0.694	0.694
Inferiorfrontal gyrus	19.233 ± 0.296	19.679 ± 0.313	1.031	0.312	0.416
Middlefrontal gyrus	40.051 ± 0.502	41.388 ± 0.530	3.23	0.076	0.152
Orbitalfrontal gyrus	23.791 ± 0.203	24.445 ± 0.214	4.706	**0.033**	0.088
Parahippocampal gyrus	3.505 ± 0.048	3.723 ± 0.058	8.791	**0.004**	**0.032**
Superiorfrontal gyrus	39.134 ± 0.641	40.803 ± 0.760	8.327	**0.005**	**0.020**

*FES* first episode schizophrenia, *HC* healthy control, *ANCOVA* controlled by age, sex and intracranial volume (ICV) and corrected for multiple comparisons among 8 regions; Bold texts indicate those with significant *p* and false discovery rate (FDR) values

### *CSF1R* fully moderated a negative association of the superior frontal gyrus with stress perception in HCs

We next explored inter-relationships among the cortical structures, CSF1R mRNA or protein, and PSS with linear regression and moderator analyses controlled by age, sex and ICV, predicting that brain structural deficit underlies stress susceptibility and CSF1R is one of the molecular machineries modulating brain and behaviour, e.g., PSSsum as the dependent variable, cortical volumes the independent variables, and CSF1R level the moderator (Fig. 2F, *n* = 46). The model showed that in the HCs but not the FES patients, the *CSF1R* mRNA was negatively associated with the PSSsum (β = -9.784, *p* < 0.05). The *CSF1R* also interacted with the superior frontal gyrus (R^2^ = 0.083, *p* < 0.05) and since the direct association of the superior frontal gyral size with the PSSsum was insignificant (β = -5.335, *p* = 0.25), this suggests that the *CSF1R* had a full moderator effect on the superior frontal gyral correlation with the PSSsum (β = 6.109, 95% confidence interval (CI) = 1.642 ~ 10.578, *p* < 0.01). Additionally, the CSF1R mRNA and protein also moderated the negative associations of the middle frontal gyrus (β = 0.383, *p* < 0.05) and the HPC (β = 0.477, *p* < 0.05) with the PSSsum in HCs, respectively (Additional file 2: Fig. S1A, *n* = 46). We also found that in the FES patients (*n* = 51), the *CSF1R* mRNA was negatively correlated with the PSSsum score (*r* = -0.249, *p* < 0.05; Additional file 2: Fig. S1B) and the PANSSt score (*r* = -0.365, *p* < 0.01; Additional file 2: Fig. S1C).

These clinical results suggest that the CSF1R might be associated with a protective response to stress-induced cortical structural changes in the HCs, which was lost in the FES patients.

### CUS and CSF1Ri enhanced anxiety in mice

**Fig. 3:**
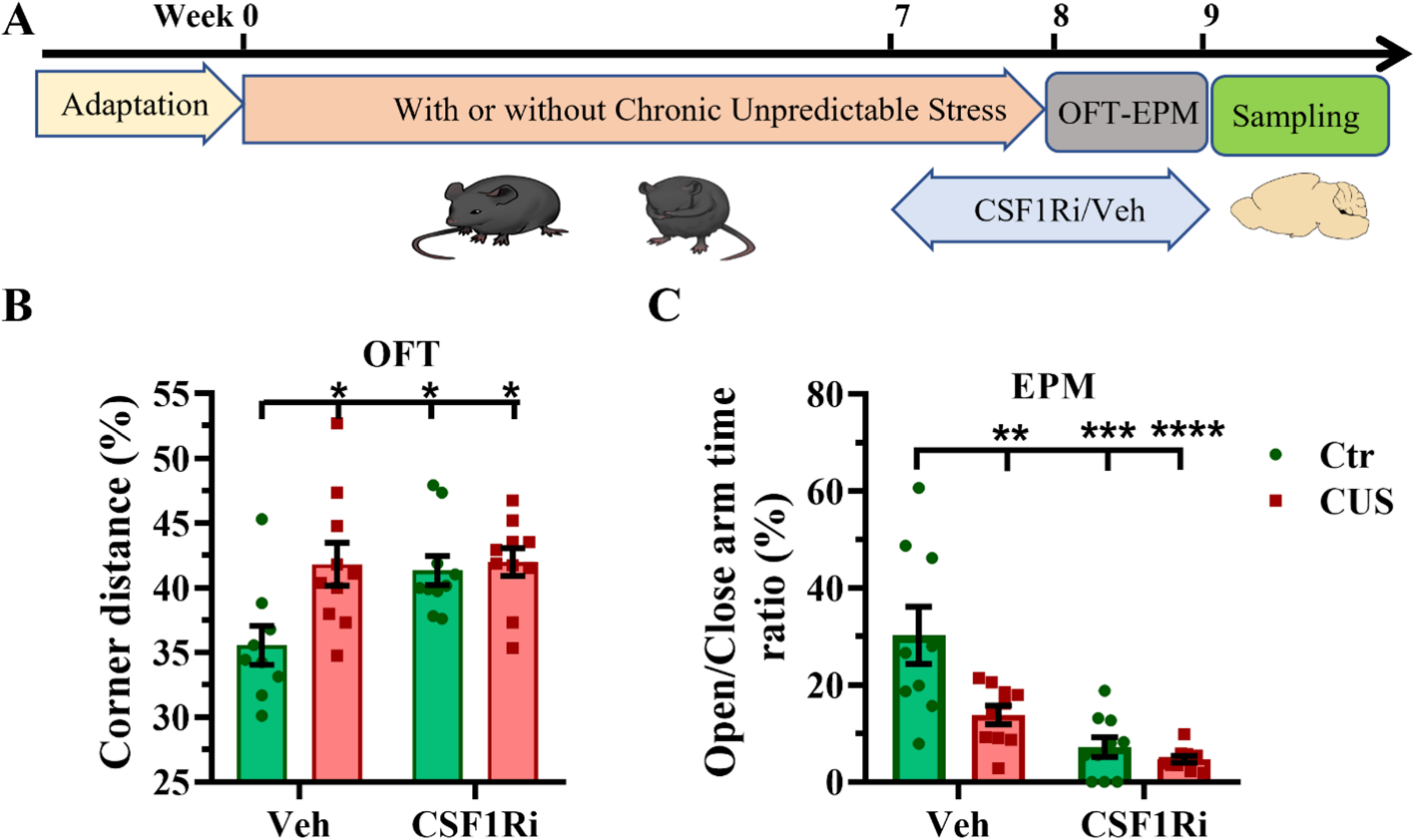
CUS and CSF1Ri enhanced anxiety in mice. (**A**) Schema representing experimental design. (**B**) ratio (%) of travel distance in corners (m) against total travel distance (m) in an open field and (**C**) ratio (%) of time spent in open arms against closed arms in an elevated plus maze (*n* = 9–10 mice per group). Ctr: Control; CUS: chronic unpredictable stress; CSF1Ri: CSF1R inhibitor; EPM: elevated plus maze; OFT: open field test; Veh: Vehicle. Data presented as mean ± SEM; */**/***/**** *p* < 0.05/0.01/0.001/0.0001 compared to Ctr-Veh. Two-way ANOVA with Bonferroni’s correction

We further applied a CUS mouse model (lasting 8 wk) combined with a CSF1Ri (3 mg PLX3397/mouse/day for 2 wk) (*n* = 9 ~ 10 mice per group) (Fig. 3A). We first evaluated their anxiety in OFT and EPM. Interactions between the CUS and the CSF1Ri on the ratio of corner distance/total distance in the OFT and the ratio of open/close arms time in the EPM were observed (both *p* < 0.001). Anxiety was enhanced by the CUS (*p* < 0.05/0.01), the CSF1Ri (*p* < 0.05/0.001), or the CUS-CSF1Ri combination (*p* < 0.05/0.0001), compared to the Ctr-Veh. No cumulative effect by the CUS-CSF1Ri combined treatment was found (Fig. 3B & 3C).

### CUS and CSF1Ri affected angiogenesis in the mouse PFC and HPC

**Fig. 4:**
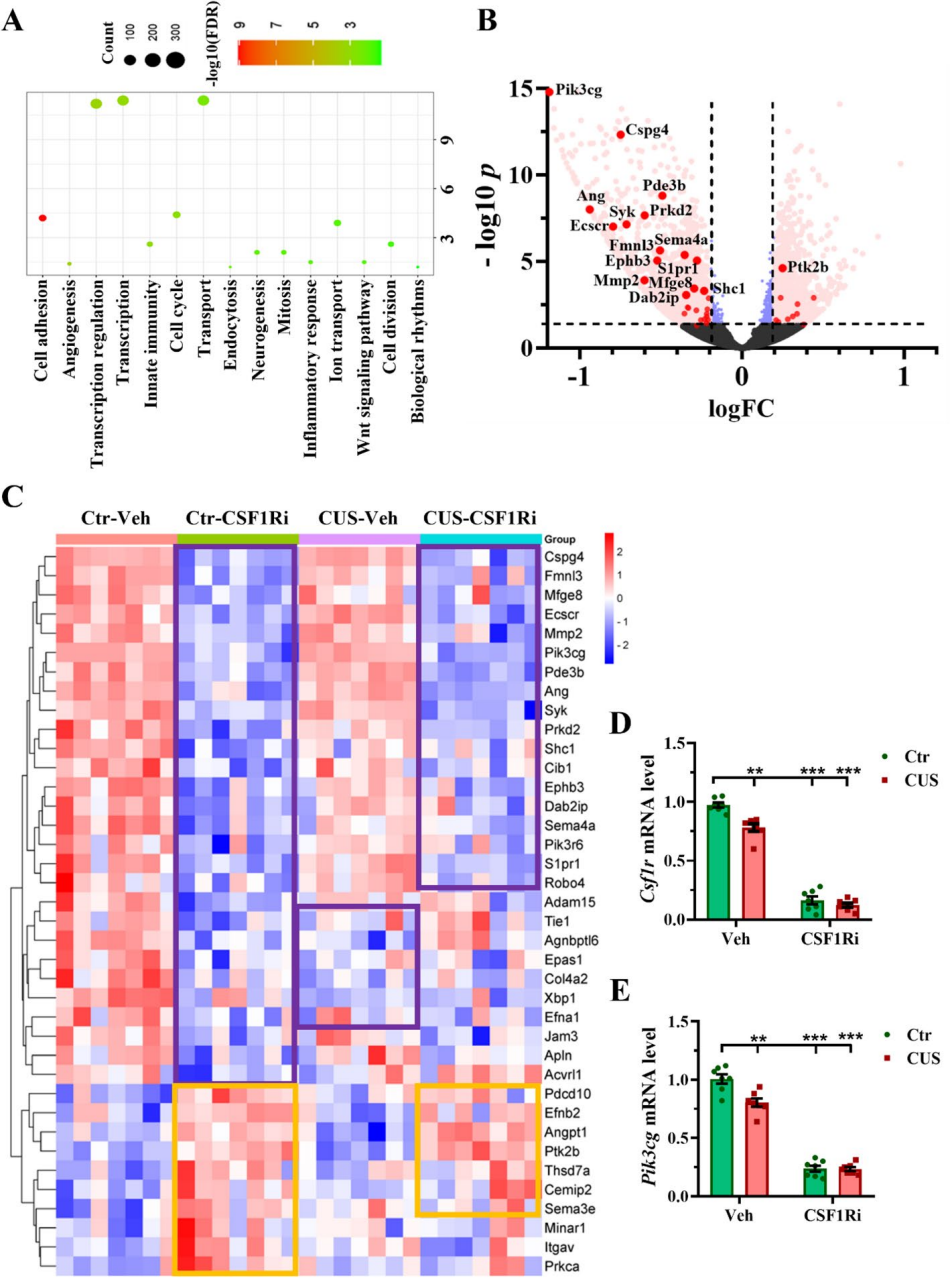
CUS and CSF1Ri inhibited *Csf1r* and angiogenic genes in mice. (**A**) GO-BP enrichment analysis shows top-ranked pathways of DEGs derived from group comparisons. (**B**) Volcano plot shows these DEGs, which are colored in red if -Log_10_ adj. *p* ≥ 1.3 and |Log_2_FC| ≥ 0.2 and in blue if |Log_2_FC| < 0.2. Angiogenic DEGs are highlighted by red dots with gene symbols when -Log_10_ adj. *p* ≥ 3. (**C**) Heatmap shows 38 angiogenic DEGs. Downregulated (in purple frame) and upregulated (in orange frame) genes are highlighted in the heatmap. (**D**) mRNA expression of *Csf1r* and (**E**) mRNA expression of *Pik3cg* in the PFC (*n* = 7 mice per group). Ctr: Control; CUS: chronic unpredictable stress; CSF1Ri: CSF1R inhibitor; Veh: Vehicle. Data presented as mean ± SEM; **/*** *p* < 0.01/0.001 compared to Ctr-Veh. Two-way ANOVA with Bonferroni’s correction. See also Additional file 1: Tables S4 & S5 and Additional file 2: Fig. S2

We next studied the PFC by RNA-seq (*n* = 7 mice per group) and identified 2750 DEGs including 1204 upregulated and 1546 downregulated DEGs among the four groups. GO-BP enrichment analysis of these DEGs showed cell adhesion and angiogenesis as the top-ranking pathways (Fig. 4A-C; Additional file 1: Tables S4 & S5; Additional file 2: Fig. S2A), with most of the angiogenic DEGs downregulated by the CSF1Ri or the CUS-CSF1Ri and a few by the CUS, compared to the Ctr-Veh (Fig. 4B & 4C). A few tight junction molecules were also downregulated by the CUS or the CSF1Ri (Additional file 2: Fig. S2A). These suggest that the CUS and the CSF1Ri affect cerebral vasculature.

To validate the RNA-seq data, we measured *Csf1r* and top-ranking angiogenic genes (*Ang*, *Cspg4*, *Pik3cg*, *Ptk2b*) in the PFC by RT-QPCR. Significant interactions existed between the CUS and the CSF1Ri on *Csf1r* and *Pik3cg* (*p* < 0.05/0.01). The CUS, the CSF1Ri, or the CUS-CSF1Ri combination reduced the *Csf1r* (Fig. 4D) and *Pik3cg* (Fig. 4E) expression, compared to the Ctr-Veh (*p* < 0.01/0.001/0.001 for both genes). The CSF1Ri also reduced the *Ang* and *Cspg4* (both *p* < 0.001) expression while enhanced the *Ptk2b* (*p* < 0.01) expression, compared to the Ctr-Veh (Additional file 2: Fig. S2B-S2D).

**Fig. 5:**
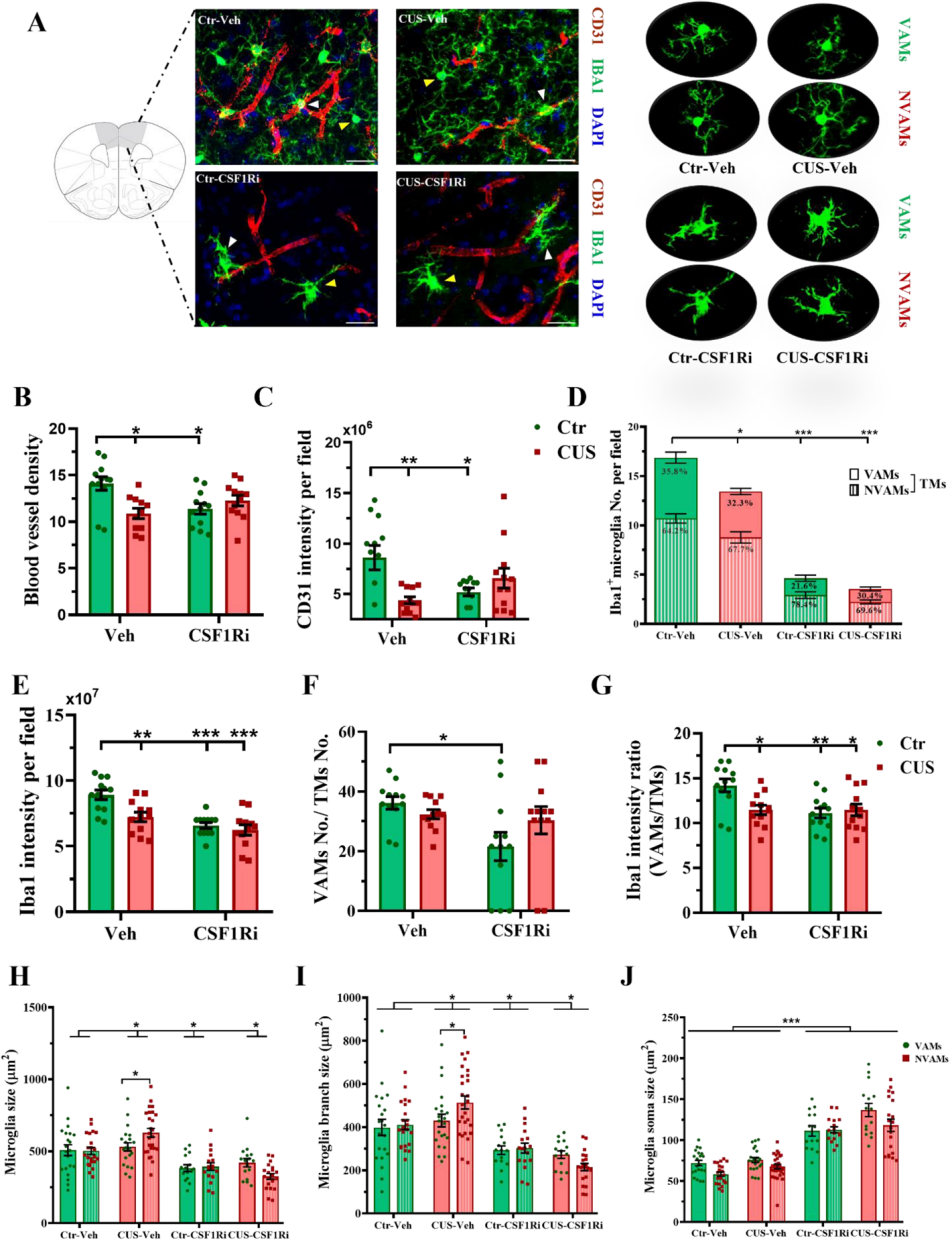
CUS and CSF1Ri reduced CD31^+^-blood vessels and differentially affected VAMs and NVAMs in mice. (**A**) Representative staining of CD31 and IBA1 in the PFC (scale bar = 10 µm) with enlarged VAMs and NVAMs (indicated by white and yellow arrowheads, respectively) are shown. (**B**) Blood vessel density (e.g., vessel area/total area*100%) and (**C**) total CD31 intensity. (**D**) TMs-(including VAMs and NVAMs)-No. and (**E**) total IBA1 intensity. (**F**) Ratio of VAMs-No./TMs-No. and (**G**) ratio of IBA1 intensity in VAMs/TMs. (**H**) Microglia cell size, (**I**) branch size and (**J**) cell soma size (*n* = 3 mice/12 sections/40 ~ 200 cells per group). Ctr: control; CUS: chronic unpredictable stress; CSF1Ri: CSF1R inhibitor; No.: number; TM: total microglia/macrophages; Veh: vehicle; VAMs: vessel-associated microglia/macrophages; NVAMs: non-vessel-associated microglia/macrophages. Data presented as mean ± SEM; */**/*** *p* < 0.05/0.01/0.001. Two-way ANOVA with Bonferroni’s correction. See also Additional file 2: Fig. S3 & S4

We further stained the PFC (*n* = 3-mice/12-sections) and HPC (*n* = 3-mice/6-sections) sections for CD31 and IBA1 by immunohistochemistry (Fig. 5A; Additional file 2: Fig. S4A). There were interactions between the CUS and the CSF1Ri on blood vessel density (PFC: *p* < 0.01, HPC: *p* < 0.05) and total CD31 intensity (both *p* < 0.01). Both parameters were decreased by the CUS or the CSF1Ri (all *p* < 0.05) compared to the Ctr-Veh, whereas no cumulative effect of the CUS-CSF1Ri combined treatment existed, compared to the Ctr-Veh or single treatment (Fig. 5B & 5C; Additional file 2: Fig. S4B & S4C). Hence, the CD31 intensity reduction was due to decreased blood vessel density, and the CUS and the CSF1Ri seemed not facilitate each other on affecting the angiogenesis.

### CUS and CSF1Ri differently affected VAMs and NVAMs in the mouse PFC and HPC

For IBA1^+^-microglia/macrophages, we classified them into VAMs (e.g., juxta-vascular microglia/macrophages) and NVAMs (e.g., non-vessel associated microglia/macrophages) among total microglia/macrophages (TMs) (PFC: *n* = 3-mice/12-sections/40 ~ 200-cells per group, HPC: *n* = 3-mice/6-sections/20 ~ 100-cells per group). Interactions between the CUS and the CSF1Ri on IBA1 intensities and cell numbers were found (all *p* < 0.05). The CUS, the CSF1Ri, or the CUS-CSF1Ri combination decreased TMs-No. (all *p* < 0.05; Fig. 5D; Additional file 2: Fig. S4D) and the total IBA1 intensity due to the loss of the TMs (all *p* < 0.05; Fig. 5E; Additional file 2: Fig. S4E), compared to the Ctr-Veh.

Although myeloid ablation by the CSF1Ri has been abundantly used in the literature, to our knowledge, only effect on total myeloid populations has been reported and very little is known about those survived microglia/macrophages that are believed to repopulate the brain as microglial progenitor cells [24, 46]. It is therefore imperative to better understand microglia/macrophage profiles in different conditions, and so we endeavoured to characterize the VAMs and NVAMs further. Interestingly, interactions between the CUS and the CSF1Ri existed on the ratios of VAMs-No./TMs-No. (PFC: *p* < 0.05) and IBA1 intensity in VAMs/TMs (both the PFC and the HPC: *p* < 0.05), which were dampened especially by the CSF1Ri in the PFC (both *p* < 0.05; Fig. 5F & 5G), and similarly in the HPC (Additional file 2: Fig. S4F & S4G), compared to the Ctr-Veh, while the ratios of the NVAMs-No./TMs-No., the IBA1 intensity in NVAMs/TMs, and the IBA1 intensity in NVAMs/VAMs were elevated (PFC: all *p* < 0.05; Additional file 2: Fig. S3A-S3C). This implies a preferential elimination of the VAMs by the CSF1Ri, possibly due to blood-route of drug delivery and/or specific sensitivity of the VAMs. The CUS or the CUS-CSF1Ri combination showed similar suppressive effect as the CSF1Ri on the ratios of IBA1 intensity, compared to the Ctr-Veh (all *p* < 0.05; Fig. 5G; Additional file 2: Fig. S3B, S3C, S4G). These results indicate that the CSF1Ri and the CUS both dampen the VAMs more preferentially than the NAVMs.

For microglial morphometrics, interactions between the CUS and the CSF1Ri on microglial cell size (the PFC: *p* < 0.05, the HPC: *p* < 0.001) and branch size were found (both *p* < 0.001). The CUS but not the other 3 conditions caused enlargement of both the microglial cell size and the branch size in the NVAMs compared to the VAMs in the PFC (both *p* < 0.05; Fig. 5H & 5I), whereas these occurred in the Ctr-Veh but no other groups in the HPC (both *p* < 0.05; Additional file 2: Fig. S4H & S4I). Combining the VAMs + NVAMs together, the CUS induced enlargement of both the microglial cell size and the branch size compared to the Ctr-Veh in the PFC (both *p* < 0.05; Fig. 5H & 5I), whereas the CSF1Ri or the CUS-CSF1Ri had opposite effects on these two parameters (all *p* < 0.05; Fig. 5H & 5I; Additional file 2: Fig. S3D & S3E). Cell soma size was grossly enlarged by the CSF1Ri compared to the Veh in the PFC (*p* < 0.001; Fig. 5J; Additional file 2: Fig. S3F). Similar morphological changes occurred in the HPC (Additional file 2: Fig. S4H-S4J).

These data overall suggest that the CSF1Ri preferentially eliminates the VAMs and de-ramifies both the VAMs and the NVAMs, and unlike the NVAMs, VAMs are resistant to the CUS-induced ramification.

### CUS decreased microglial abundancy and dampened microglial Csf1r expression in the mouse HPC

**Fig. 6:**
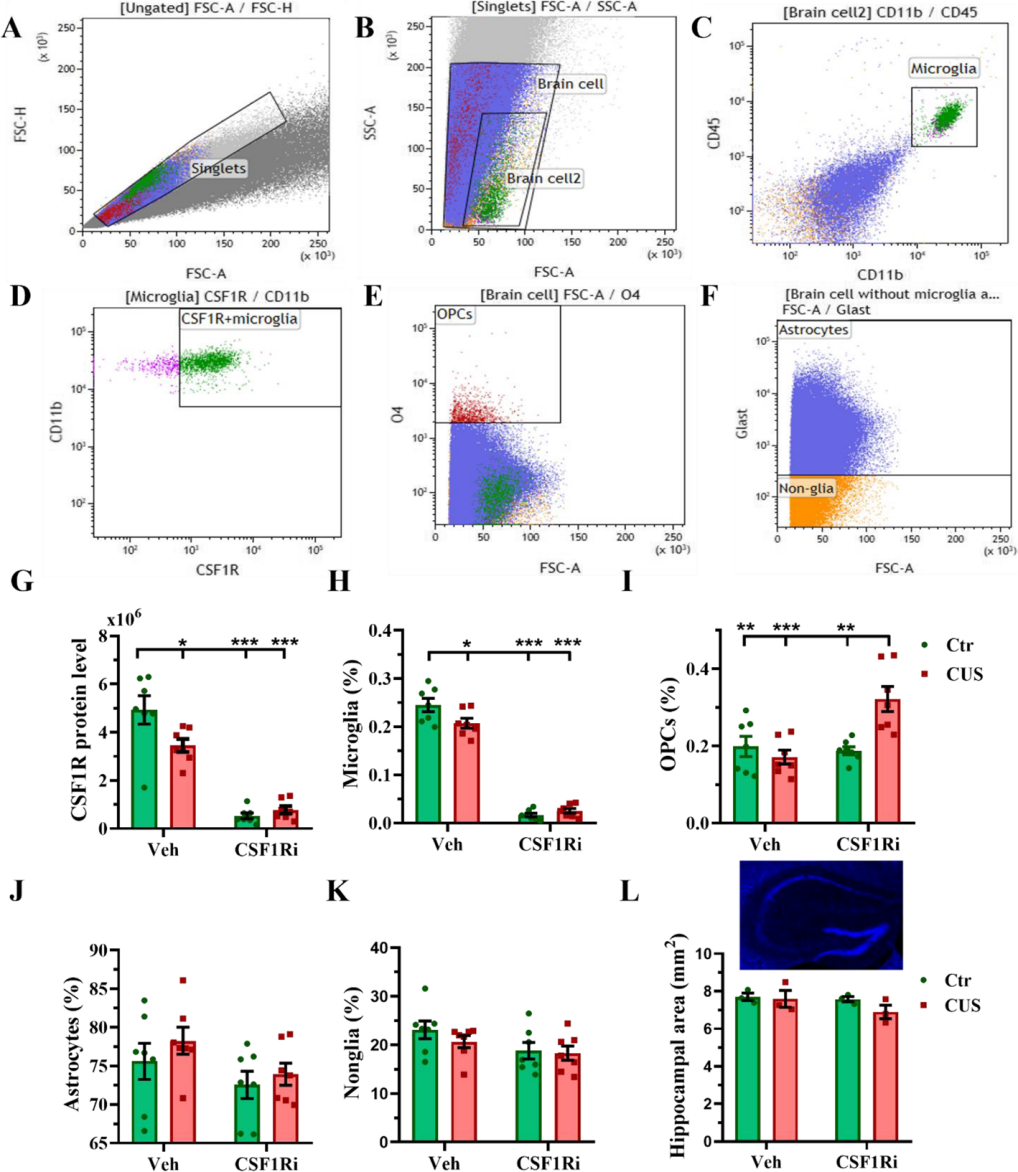
CUS and CSF1Ri reduced microglia and Csf1r level in mice. (**A**-**F**) Representative flow cytometry dot plots showing gating strategy for hippocampal microglia, Csf1r^+^-microglia, OPCs, astrocytes, and nonglia. (**G**) Csf1r protein level expressed by total microglia. (**H**–**K**) % of total microglia, OPCs, astrocytes, and nonglia (*n* = 7 mice per group). (**L**) Hippocampal area (*n* = 3 mice per group). Ctr: control; CUS: chronic unpredictable stress; CSF1Ri: CSF1R inhibitor; No.: number; OPC: oligodendrocyte precursor cell; Veh: vehicle. Data presented as mean ± SEM; */**/*** *p* < 0.05/0.01/0.001. Two-way ANOVA with Bonferroni’s correction

We further validated CSF1Ri by quantifying microglia along with other glial populations, namely astrocytes, OPCs, and nonglia in the HPC by flow cytometry (*n* = 7 mice per group). A hierarchical gating strategy is shown by representative dot plots in Fig. 6A-F and negative staining by isotype control antibodies is shown in Additional file 2: Fig. S5.

Remarkably, like the TMs in the PFC (Fig. 5D & 5E), there were interactions between the CUS and the CSF1Ri on percentage (%) of total hippocampal microglia and total Csf1r protein level (both *p* < 0.05). The CUS,  the CSF1Ri, or the CUS-CSF1Ri combination reduced the total Csf1r protein (due to loss of microglia and in line with RNA-seq/QPCR results, Fig. 6G) and the microglia% (Fig. 6H) compared to the Ctr-Veh (*p* < 0.05/0.001/0.001 for both parameters). Furthermore, measuring MFI of Csf1r on each survived microglia, we observed its significant decrease induced by the CUS compared to the Ctr-Veh (*p* < 0.01; Additional file 2: Fig. S2E), recapitulating our clinical data.

Interestingly, we observed an additional interaction of the CUS and the CSF1Ri (*p* < 0.01) on the OPCs. The CUS-CSF1Ri jointly increased the OPCs compared to the Ctr-Veh, the CUS-Veh, or the Ctr-CSF1Ri (*p* < 0.01/0.001/0.01), while single treatment didn’t affect the OPCs compared to the Ctr-Veh, indicating a boosting effect of the CUS-CSF1Ri combination on the OPCs compared to single treatment (Fig. 6I). Nevertheless, the CUS and the CSF1Ri didn’t affect the abundancy of the astrocytes and nonglia (Fig. 6J & 6K). We also measured area of the HPC (*n* = 3 mice per group) stained by DAPI in immunohistochemistry. The CUS and the CSF1Ri didn’t affect this measure (Fig. 6L).

## Discussion

The current study shows that FES patients perceived higher stress than HCs; CSF1R level and PFC subregional size were decreased in the FES patients; *CSF1R* mRNA level was associated with shrinkage of the superior frontal gyrus in response to perceived stress in the HCs but not FES patients; Similar to CUS, CSF1Ri enhanced anxiety and downregulated angiogenesis in mice; The CSF1Ri preferentially eliminated VAMs and induced cytoarchitectural changes differently than the CUS in the mouse brain. These are to our knowledge the first evidence revealing the involvement of CSF1R in schizophrenia and vascular association of microglia/macrophages in the context of stress.

Our finding of lower *CSF1R* mRNA and protein in the FES patients is in line with several previous studies. Lower level of *CSF1R* mRNA was reported in the cortices [31–33] and spleens of chronic schizophrenia patients [47]. The reduction of *CSF1R* observed in these studies can be affected by disease chronicity or anti-psychotics. By contrast, our current observation on the blood CSF1R level had minimal drug effect, giving hint on impairment of the CSF1R function in early stage of schizophrenia. Given that the CSF1R is almost exclusively expressed by myeloid cells in the brain, and changes in the cerebrovascular permeability have been identified in psychiatric disorders, thereby allowing peripheral inflammatory impact on the brain [48], our findings may be highly relevant for stress-induced brain pathophysiology of the FES.

We found that a PFC subarea, the superior frontal gyrus, was smaller in the FES patients than the HCs. Although there was no structural change in the HPC, we observed smaller volumes of the neighboring parahippocampal gyrus in the FES patients than  the HCs. Deficits of these regions have been associated with psychotic symptoms such as auditory hallucinations and disordered thoughts in schizophrenia [49, 50]. More interestingly, we found that volumes of some of the PFC subregions and the HPC were negatively associated with PSS. Furthermore, the blood CSF1R mRNA or protein interacted with these brain regions and moderated their negative associations with the PSS in the HCs but not the FES patients. Additionally, the CSF1R mRNA level was also negatively correlated with both the PSS and the PANSSt scores. These suggest the importance of the CSF1R in stress regulation via modulating these limbic structures, which might be dysfunctional in the FES patients. Besides, the PSS scores were positively correlated with the PANSSt, supporting the notions that stress exacerbates psychosis [4] and severity of psychotic symptoms correlates with that of anxiety symptoms [51].

To depict how the CSF1R may regulate stress response, we used a CUS mouse model and administered an inhibitor (PLX3397, CSF1Ri) to block Csf1r in microglia. Importantly, we found that the CSF1Ri was anxiogenic to B6N mice similarly as the CUS. Our observation corroborates with previous studies reporting that *Csf1r*^+/−^ mice exhibited anxiety along with cognitive and sensorimotor deficit [25]. However, other studies have not observed effect of the CSF1Ri on the anxiety in mice [24]. These discrepancies may be due to different ways of the CSF1Ri administration, which warrants further careful investigations.

We found that IBA1 intensity in VAMs were more sensitively dampened by the CUS compared to NVAMs, implicating a less juxta-vascular association of microglial/macrophage processes after the CUS. Meanwhile, the CUS decreased microglia in the PFC and the HPC, which may be explained by both our own observation that the CUS dampened microglial Csf1r expression, which is pivotal for microglial survival, and a previous report that the CUS induced microglial apoptosis [30]. Our observation on the dampened Csf1r expression is also in line with an earlier study on the CUS [52] and supports our clinical data showing that the lower CSF1R was associated with the higher PSS. It should be cautioned that the VAMs may also constitute perivascular macrophages, which adopt microglial phenotype after the CUS or the CSF1Ri and are challenging to discern as they share common myeloid markers with microglia, including the Csf1r and IBA1 [15].

Intriguingly, the CSF1Ri did not facilitate the CUS’s effect on anxiety behaviours and microglial parameters, including the number and morphology of the VAMs, as well as the expressions of CD31 and some angiogenic genes. We speculate that since the CUS dampened both microglial abundancy and Csf1r level in microglia, this made stressed microglia less sensitive to the CSF1Ri. Nevertheless, some brain cytoarchitecture such as OPCs may be robust in stress adaptation due to their regenerative capacity, which might be ignited by the CSF1Ri, as we observed here (Fig. 6I). The desensitization of microglial Csf1r after the CUS also notably corroborates with our clinical observation that the negative association of the *CSF1R* level with the PSS was less significant in the FES patients compared to the HCs.

Chronic stress affects angiogenesis by dampening endothelial molecules and accelerating vascular inflammation [53] and reduced angiogenesis is implicated in stress-related psychiatric disorders [48, 54]. Stress also compromises the blood–brain barrier in psychiatric conditions [54, 55] and changed gene expression of brain endothelial cell adhesion molecules in schizophrenia patients with “high inflammation” [56]. Our RNA-seq and immunohistochemistry findings on the CUS-compromised angiogenesis in the mouse PFC are consistent with these previous studies and suggest that the blood vessel-association of microglia/macrophages may regulate cerebrovascular integrity.

Importantly, we also found that the CSF1Ri downregulated most of the cell adhesion and angiogenic DEGs such as *Pik3cg*, decreased blood vessel density, and preferentially diminished the juxta-vascular VAMs in the mouse PFC. Corroboratively, a recent study reported CSF1Ri-induced blood–brain barrier leakage via dampened tight junction genes [57]. Little is known about microglia-vascular interactions in the adult brain, however. One recent study found that about 30% of microglia are capillary-associated and constantly survey the influx of blood-borne components into living adult mice; furthermore, microglial depletion with the CSF1Ri (PLX3397) induced a 15% increase in capillary diameter compared to control [58]. Our RNA-seq and immunohistochemistry findings on the CSF1Ri support this in vivo imaging study and provide a further depiction of molecular mechanisms on microglial association with neurovascular unit. Currently, clinical and preclinical studies on angiogenic function of microglia/macrophages in psychiatric conditions are still missing, our work thereby gives a first glimpse into this theme.

Our study has several limitations to mention. These include the small sample size in our clinical and preclinical cohorts, no comparison on potential sex difference (only male mice were studied), and the cross-sectional nature of our clinical study design. Additionally, the PSS used in our current study can only reflect perceived stress during past one month of a subject, while CTQ score that reflects early life trauma did not show significant results in the FES patients, indicating it not as the sole determinant of psychosis. This suggests that including other clinical parameters that reflect longer adulthood traumatic experiences, such as life event scale (LES) for the past one year and hair cortisol content for the past three months, may make the results more accurate and convincing.

## Conclusions

Our findings suggest that CSF1R may provide a stress-coping mechanism via regulating microglial/macrophagic association with cerebral vasculature, which might be disturbed in schizophrenia. Currently, only generic anti-inflammatory drugs have been tried in clinical psychiatric studies, with limited and even debatable therapeutic effects. This calls for a better understanding of functions of microglial subpopulations and their effector molecules, which would provide more specific candidate targets for developing diagnostic biomarkers and therapeutic drugs in neuropsychiatric disorders. Our findings may hence be helpful for developing such tools to tackle these disorders.

### Supplementary Information

**Additional file 1: Table S1.** List of mouse gene qPCR primers. **Table S2.** Human blood DEGs_FES/HC. **Table S3.** Human blood DEGs_GOBP. **Table S4.** Mouse PFC DEGs_CUS/CSF1Ri. **Table S5.** Mouse PFC DEGs_GOBP.

**Additional file 2: Fig S1. **CSF1R facilitated the negative associations of brain structures with PSS scores in HCs. **Fig S2.** CUS/CSF1Ri affected expression of DEGs mediating cell adhesion and Csf1r in the mouse PFC. **Fig S3.** Changes of IBA1 intensity and morphology in microglia/macrophages by CUS and CSF1Ri treatments in the mouse PFC. **Fig S4.** CUS/CSF1Ri reduced CD31+-blood vessels and differentially affected VAMs and NVAMs in the mouse HPC. **Fig S5.** Representative dot plots of negative controls used in flow cytometric analysis.

## Data Availability

The RNA-seq datasets presented in this study can be found in the European Nucleotide Archive (ENA) repository with the accession number: PRJEB53454.

## Abbreviations

ANOVA: Analysis of variance
ANCOVA: Analysis of covariance
CSF1R: Colony stimulating factor 1 receptor
CSF1Ri: CSF1R inhibition
Ctr: Control
CUS: Chronic unpredictable mild stress
CTQ: Childhood trauma questionnaire
DEGs: Differentially expressed genes
EPM: Elevated plus maze
FDR: False discovery rate
FES: First episode schizophrenia
GO: Gene ontology
HC: Healthy controls
ICV: Intracranial volume
MFI: Mean fluorescent intensity
MRI: Magnetic resonance imaging
No.: Number
NVAMs: Nonvessel-associated microglia/macrophages
OFT: Open field test
OPC: Oligodendrocyte precursor cell
PANSSt: Positive and negative symptom scale-total score
PPI: Protein–protein interaction
PSSsum: Perceived stress scale summation score
RNA-seq: RNA sequencing
TMs: Total microglia/macrophages
Veh: Vehicle
VAMs: Vessel-associated microglia/macrophages
