# Supplementary material for: CSF1R regulates schizophrenia-related stress response and vascular association of microglia/macrophages
Source: BMC Med. 2023 Aug 4;21:286. doi: 10.1186/s12916-023-02959-8 (PMC10403881; doi:10.1186/s12916-023-02959-8)
Supplement: Supplementary file 1 — Additional file 1: Table S1. List of mouse gene qPCR primers. Table S2. Human blood DEGs_FES/HC. Table S3. Human blood DEGs_GOBP. Table S4. Mouse PFC DEGs_CUS/CSF1Ri. Table S5. Mouse PFC DEGs_GOBP. [file 12916_2023_2959_MOESM1_ESM.pdf]

Table S1: mouse gene qPCR primers

Gene (mouse)	Primer	Sequence
Ang	Forward	CCAACAGGAAGGAAGGAGTGA
	Reverse	CTGGGCTTATCGCCATCTCTT
Csf1r	Forward	TGGCGAGGGTTCATTATCCG
	Reverse	CCAGCTTGCTAGGCTCCAAT
Cspg4	Forward	TAGGGAGCAGGCAAACGAAG
	Reverse	AAACTCAAACGACGCACAGC
Pik3cg	Forward	GAAGGGAGCCCCAGAAAAGAG
	Reverse	GTGATGCGGAGGAGGATCATT
Ptk2b	Forward	CTACACGGAGTTCACAGGGC
	Reverse	TACACGAGGTCATCGGTCCT
Actin	Forward	ACTGAGCTGCGTTTTACACCC
	Reverse	GCCTTCACCGTTCCAGTTTT

Table S2: human blood DEGs

Symbols	logFC	P.Value	adj.P.Val
FGF17	0,727	3,48E-06	1,03E-05
CTSK	0,601	2,23E-11	2,95E-10
APOE	0,524	0,000789	0,00156
NOTCH1	0,4	1,10E-07	4,39E-07
NDST1	0,35	4,10E-05	0,000101
AKT1	0,345	4,84E-07	1,68E-06
MAFB	0,321	7,77E-05	0,000182
GNA11	0,321	6,89E-07	2,31E-06
STAT5B	0,25	4,45E-06	1,29E-05
KAT6A	0,174	0,005764	0,00988
STAT3	0,168	0,002891	0,005193
CSF1R	-0,195	0,001794	0,003335
PIK3CA	-0,201	0,004873	0,008441
GNAS	-0,21	0,002965	0,00531
MITF	-0,298	0,002515	0,004565
F13A1	-0,319	0,002746	0,004952
SLC26A2	-0,322	5,90E-05	0,000141
SLC7A7	-0,333	7,96E-08	3,28E-07
GNAQ	-0,358	1,44E-05	3,81E-05
PNP	-0,39	2,01E-08	9,57E-08
NFIA	-0,485	6,34E-08	2,68E-07
CFH	-0,497	1,02E-05	2,79E-05
TLR3	-0,509	7,03E-08	2,93E-07
PTEN	-0,516	1,54E-10	1,44E-09
ITGB3	-0,536	0,000228	0,000492
TNFSF11	-0,721	2,64E-05	6,70E-05
EGF	-0,82	1,54E-07	5,95E-07
NPM1	-0,858	6,05E-11	6,63E-10

Table S3: human blood DEGs_GOBP

Pathway	Genes	FDR
regulation of developmental process	GNA11, APOE, PIK3CA, STAT3, EGF, CTSK, NOTCH1, CSF1R, GNAQ, STAT5B, MITF, TLR3, PNP, GNAS, PTEN, MAFB, TNFSF11, AKT1, ITGB3	3,64E-08
regulation of cell differentiation	GNA11, APOE, STAT3, CTSK, NOTCH1, GNAQ, STAT5B, MITF, TLR3, PNP, GNAS, PTEN, MAFB, TNFSF11, AKT1, ITGB3	1,63E-07
regulation of protein phosphorylation	APOE, NDST1, PIK3CA, STAT3, EGF, NOTCH1, CSF1R, GNAQ, TLR3, NPM1, PTEN, TNFSF11, AKT1, ITGB3	1,02E-06
transmembrane receptor protein tyrosine kinase signaling pathway	NDST1, PIK3CA, STAT3, EGF, CSF1R, STAT5B, FGF17, PTEN, AKT1, ITGB3	1,02E-06
multicellular organismal process	GNA11, APOE, NDST1, PIK3CA, STAT3, F13A1, EGF, CTSK, NOTCH1, SLC26A2, CSF1R, GNAQ, STAT5B, MITF, TLR3, FGF17, PNP, GNAS, NFIA, PTEN, MAFB, KAT6A, TNFSF11, AKT1, ITGB3	1,02E-06
system development	GNA11, APOE, NDST1, PIK3CA, STAT3, EGF, NOTCH1, CSF1R, GNAQ, STAT5B, MITF, TLR3, FGF17, GNAS, NFIA, PTEN, MAFB, KAT6A, TNFSF11, AKT1, ITGB3	1,02E-06

Table S4: mouse PFC DEGs

Celladhesion				Angiogenesis			
Symbols	logFC	P.Value	adj.P.Val	Symbols	logFC	P.Value	adj.P.Val
Selplg	-1,35	4,33E-20	1,83E-17	Pik3cg	-1,19	5,23E-18	1,59E-15
Itgam	-1,27	5,26E-18	1,59E-15	Cspg4	-0,75	2,09E-15	4,67E-13
Csf3r	-1,02	2,70E-12	3,07E-10	Pde3b	-0,49	1,67E-11	1,58E-09
Itgb5	-0,71	1,89E-14	3,42E-12	Ang	-0,94	1,29E-10	9,99E-09
Negr1	0,44	3,31E-13	4,63E-11	Prkd2	-0,60	3,05E-10	2,14E-08
Myh9	-0,36	9,58E-13	1,23E-10	Syk	-0,71	1,20E-09	7,13E-08
Parvg	-0,77	6,75E-12	6,99E-10	Ecscr	-0,80	1,72E-09	9,61E-08
Atp1b1	0,29	6,44E-11	5,44E-09	Fmnl3	-0,51	6,22E-08	2,27E-06
Prkd2	-0,60	3,05E-10	2,14E-08	Sema4a	-0,35	1,28E-07	4,20E-06
Cntn1	0,29	3,88E-10	2,66E-08	S1pr1	-0,28	2,99E-07	8,72E-06
Pxn	-0,36	4,55E-09	2,34E-07	Ephb3	-0,52	3,01E-07	8,75E-06
Cdh2	0,31	4,58E-09	2,34E-07	Ptk2b	0,25	9,81E-07	2,44E-05
Siglece	-0,85	5,29E-09	2,61E-07	Mmp2	-0,60	6,53E-06	0,000122
Pcdha6	0,70	6,40E-08	2,33E-06	Mfge8	-0,29	2,37E-05	0,000358
Cd33	-0,75	8,89E-08	3,10E-06	Shc1	-0,23	3,46E-05	0,000491
Arvcf	-0,31	9,61E-08	3,32E-06	Dab2ip	-0,35	6,81E-05	0,000858
Ackr3	-0,41	1,04E-07	3,51E-06	Thsd7a	0,44	0,000106	0,001224
Cdh9	0,50	1,37E-07	4,47E-06	Pdcd10	0,24	0,000108	0,001248
Gp1bb	-0,48	1,47E-07	4,75E-06	Adam15	-0,21	0,000116	0,001332
Kitl	0,34	1,81E-07	5,65E-06	Prkca	0,34	0,000297	0,002886
Lpxn	-0,72	3,05E-07	8,82E-06	Angptl6	-0,34	0,000492	0,00435
Ctnnd1	-0,26	4,76E-07	1,31E-05	Apln	-0,33	0,000552	0,004776
Pcdhga2	0,37	1,90E-06	4,32E-05	Col4a2	-0,21	0,000678	0,005655
Pcdhga4	0,38	2,92E-06	6,25E-05	Cib1	-0,28	0,000809	0,006503
Cntnap2	0,38	4,26E-06	8,61E-05	Pik3r6	-0,36	0,001398	0,010071
Itga4	0,39	6,09E-06	0,000115	Angpt1	0,34	0,001471	0,010477
Flrt3	0,41	6,69E-06	0,000124	Xbp1	-0,22	0,00148	0,010534
Pcdhac2	0,29	8,32E-06	0,000148	Sema3e	0,31	0,002255	0,014593
Astn1	0,25	1,15E-05	0,000195	Epas1	-0,22	0,002337	0,014995
Nuak1	-0,38	1,21E-05	0,000202	Cemip2	0,28	0,003651	0,021271
Omg	0,25	1,40E-05	0,000229	Tie1	-0,23	0,003734	0,02169
Pcdha7	0,54	1,53E-05	0,000249	Jam3	-0,21	0,00417	0,023665
Cntn6	0,34	1,73E-05	0,000275	Efnb2	0,21	0,004281	0,024089
Mfge8	-0,29	2,37E-05	0,000358	Robo4	-0,25	0,004358	0,024386
Trip6	-0,37	2,78E-05	0,000409	Itgav	0,23	0,00599	0,031509
Cadm2	0,24	2,82E-05	0,000413	Acvrl1	-0,22	0,007264	0,036716
Pcdhgb6	0,35	2,92E-05	0,000427	Minar1	0,38	0,00889	0,042918
Cd84	-0,60	3,59E-05	0,000508	Efna1	-0,28	0,01063	0,049248
Vcl	-0,24	4,08E-05	0,000559				
Lgals3bp	0,58	4,11E-05	0,00056				
Fermt3	-0,59	4,36E-05	0,000585				
Hepacam	-0,23	8,05E-05	0,000983				
Nlgn1	0,27	8,89E-05	0,001068				
Chl1	0,34	9,63E-05	0,001142				
Adam15	-0,21	0,000116	0,001332				
Dscaml1	0,33	0,000126	0,001422				
Reln	0,45	0,000139	0,001549				

Itga8	0,36	0,000143	0,001588
Cd47	0,25	0,000154	0,001685
Mslnl	-0,50	0,000157	0,001713
Cntn4	0,23	0,000171	0,001831
Flrt2	0,33	0,000219	0,002262
Itga9	-0,39	0,000241	0,002451
Pcdhga5	0,24	0,000249	0,002518
Pcdhgc5	0,25	0,000255	0,002563
Prkca	0,34	0,000297	0,002886
Fat3	0,29	0,000299	0,002895
Nrcam	0,21	0,000334	0,003167
Pcdhb17	0,24	0,000349	0,003286
Nphs1	-0,49	0,00035	0,00329
Lamb1	0,34	0,000358	0,003344
Col16a1	-0,21	0,000365	0,003397
Pcdha5	0,43	0,000388	0,003574
Cd36	-0,49	0,0004	0,00366
Pcdhb11	0,41	0,000588	0,005003
Ssx2ip	0,22	0,0006	0,005099
Tenm3	0,50	0,000643	0,005408
Cib1	-0,28	0,000809	0,006503
Cntnap5a	0,42	0,000839	0,006706
Lama1	0,47	0,0009	0,007093
Tyro3	-0,22	0,000904	0,007119
Inppl1	-0,24	0,000955	0,007433
Cntn5	0,43	0,001037	0,007934
Pcdhb22	0,32	0,001069	0,008122
Cdh8	0,36	0,001078	0,00817
Pcdhb14	0,33	0,00123	0,009067
Itgb2	-0,46	0,00129	0,009408
Postn	0,43	0,001426	0,010228
Ccn1	-0,45	0,001471	0,010477
Pcdh15	-0,32	0,00157	0,011037
Cd44	0,38	0,001837	0,012509
Pcdhb12	0,30	0,001876	0,012711
Edil3	0,20	0,002202	0,014338
Pcdhga12	0,21	0,002227	0,014457
Pcdhb18	0,27	0,002318	0,014919
Col13a1	0,44	0,002371	0,015144
Cntnap3	0,43	0,002402	0,015308
Spp1	0,44	0,002586	0,016242
Cdh13	0,31	0,002884	0,017683
Pcdhga9	0,28	0,00291	0,017797
Pip5k1c	-0,26	0,003179	0,019156
Pcdh17	0,32	0,0032	0,019244
Scarf1	-0,28	0,003462	0,020399
Pcdhb21	0,43	0,003512	0,020617
Pcdhga10	0,27	0,003516	0,020631
Pcdhgb1	0,28	0,004054	0,023167
Jam3	-0,21	0,00417	0,023665

Pcdhb7	0,23	0,004593	0,02548
Pcdhb20	0,22	0,005107	0,027715
Cntnap4	0,31	0,005176	0,028018
Tnr	0,35	0,005803	0,030717
Itgav	0,23	0,00599	0,031509
Pcdh19	0,28	0,006454	0,033415
Ncam2	0,39	0,006651	0,034224
Cgref1	0,27	0,006995	0,035671
Col15a1	0,38	0,0076	0,037997
Pcdha3	0,33	0,007668	0,038263
Col6a4	0,39	0,007808	0,038783
Siglecg	-0,26	0,007958	0,03937
Amigo3	-0,22	0,009125	0,04373
Alcam	0,22	0,009252	0,044179
Boc	-0,28	0,009703	0,045895
Cdh12	0,27	0,010084	0,047293
Comp	-0,35	0,010159	0,047562
Pcdha9	0,30	0,010553	0,048935

Table S5: mouse PFC DEGs_GOBP

Pathway	Genes	FDR
Cell adhesion	CD84, COL16A1, CSF3R, CTNND1, INPPL1, CIB1, CDH9, CDH8, COMP, ALCAM, CDH2, CNTNAP5A, TNR, NRCAM, CCN1, PCDHAC2, POSTN, OMG, PRKCA, ATP1B1, ADAM15, TYRO3, PRKD2, VCL, ASTN1, COL13A1, HEPACAM, PXN, PCDH15, PCDH19, PCDH17, NUAK1, FLRT2, FLRT3, PCDHA5, LPXN, PCDHA3, PIP5K1C, PCDHA9, PCDHA7, PCDHA6, CNTN5, CNTN6, CADM2, PCDHGA10, PCDHGA12, PARVG, KITL, TRIP6, CNTN1, FAT3, CNTN4, FERMT3, PCDHB7, CNTNAP3, LGALS3BP, CNTNAP2, PCDHGB6, ITGAM, TENM3, ITGB5, ITGB2, SIGLECG, SIGLECE, CHL1, BOC, ITGAV, CD36, EDIL3, SCARF1, CD33, PCDHGA5, ITGA4, PCDHGA4, GP1BB, PCDHGA2, PCDHGA9, PCDHGB1, ITGA8, CDH12, COL6A4, CDH13, MYH9, CD47, MFGE8, SSX2IP, DSCAML1, CD44, CNTNAP4, ITGA9, COL15A1, NLGN1, SELPLG, AMIGO3, LAMA1, MSLNL, PCDHB22, PCDHB21, PCDHB20, ARVCF, RELN, SPP1, NCAM2, JAM3, CGREF1, NEGR1, PCDHGC5, PCDHB14, LAMB1, PCDHB12, PCDHB11, NPHS1, PCDHB18, PCDHB17, ACKR3	6,2E-10
Angiogenesis	ACVRL1, ROBO4, SHC1, EPAS1, PDE3B, ECSCR, CIB1, SEMA3E, PIK3R6, PIK3CG, EFNB2, PDCD10, S1PR1, PTK2B, ITGAV, THSD7A, EPHB3, JAM3, MINAR1, SEMA4A, XBP1, ANGPT1, SYK, CEMIP2, TIE1, MMP2, DAB2IP, PRKCA, APLN, EFNA1, FMNL3, COL4A2, ADAM15, ANGPTL6, ANG, PRKD2, CSPG4, MFGE8	3,6E-04

Transcription regulation	RB1, SPI1, MYT1L, CCNH, NEXMIF, SCX, IKZF1, SOX21, ELK3, SOX3, SOX17, SOX18, GPBP1, DPF1, KAT7, SOX8, CCNL2, IER5, JUNB, MEF2A, SP110, SOX13, RFX3, EBF3, RFX1, SOX10, OVOL2, RUNX1, JMJD6, ZFP292, DDIT3, XRN2, BTK, TXNIP, BCORL1, ZFPM1, L3MBTL1, DHX9, EPAS1, ZFP507, HIF1A, DEDD2, MIER2, DHX36, HIVEP1, ZSCAN12, ZBTB7B, PPARGC1A, BANP, MCTS1, EGR1, EGR2, XBP1, JUND, EGR3, EGR4, BCL11B, FOXF2, NFATC3, TRIP4, IRF2BP2, NFATC1, NR1D1, SMARCA1, PBX1, FLI1, NR2F6, POU6F1, HNRNPK, ZBTB6, ID2, ID1, TRIP6, ID3, NOL11, IFI27L2A, MESP2, CSRNP1, TENM1, RARG, ZFP488, ELL, BHLHE41, LIMD1, GLI1, GLI2, ZFP385A, ZFP366, PBRM1, BATF3, NCOA3, PROX2, IL16, PPHLN1, SIRT7, BAZ1A, PPRC1, CBFA2T3, ZHX1, SIRT2, MN1, TOX3, ZEB2, MED25, RARA, NCOA7, PPARA, PPARD, ELOF1, RELA, TCEAL7, PURA, SERTAD3, BCLAF1, SERTAD1, SERTAD2, TCEAL1, LEO1, SUZ12, ZFP710, PCGF6, STAT1, PCGF2, NR1H2, YLPM1, UNCX, LRPPRC, GATAD2A, PER2, PER1, NR6A1, NFIC, TCEA1, PKN1, SUPT16, ZFP276, CRTC2, NAB2, CTNND1, HR, ETS1, LOXL2, NR3C2, FOXQ1, CCND3, KMT5A, ZFP37, ZFP39, ZFP579, TLE3, ZFP335, TLE2, ZGPAT, ZFP219, KMT5C, LMO3, ARID5A, ZBTB33, GTF2F2, POU3F3, RSBN1, HIC1, TLE6, NPAS4, HIC2, MAF, PSPC1, KAT6B, ZFP692, ZFP691, LRIF1, KHDRBS1, KHDRBS2, AKNA, CREM, FOXO6, FOXO4, NPAS1, GTF2E1, GLIS2, ATXN3, ZKSCAN8, ZKSCAN3, SAFB2, NLRP3, LPXN, E4F1, TFAP2D, ZFHX2, CBX3, SMAD9, ESRRG, POU2F2, ESR1, ZFHX4, HOPX, NFKB1, SCRT1, SMAD7, NR4A1, MLXIPL, MRGBP, NR4A3, MAFB, SP2, CNOT1, LHX2, SP4, BHLHE40, NFE2L2, MXD4, PHF19, RNF10, ZFP523, KDM5D,	3,6E-04

Transcription	RB1, SPI1, MYT1L, CCNH, NEXMIF, SCX, IKZF1, SOX21, ELK3, SOX3, SOX17, SOX18, GPBP1, DPF1, KAT7, SOX8, CCNL2, IER5, JUNB, MEF2A, SP110, SOX13, RFX3, EBF3, RFX1, SOX10, OVOL2, RUNX1, JMJD6, ZFP292, DDIT3, XRN2, BTK, TXNIP, BCORL1, ZFPM1, L3MBTL1, DHX9, EPAS1, ZFP507, HIF1A, DEDD2, MIER2, HLTF, DHX36, HIVEP1, ZSCAN12, ZBTB7B, PPARGC1A, BANP, MCTS1, EGR1, EGR2, XBP1, JUND, EGR3, EGR4, BCL11B, FOXF2, NFATC3, TRIP4, IRF2BP2, NFATC1, NR1D1, SMARCA1, PBX1, FLI1, NR2F6, POU6F1, HNRNPK, ZBTB6, ID2, ID1, TRIP6, ID3, NOL11, IFI27L2A, MESP2, CSRNP1, TENM1, RARG, ZFP488, ELL, BHLHE41, LIMD1, GLI1, GLI2, ZFP385A, ZFP366, PBRM1, BATF3, NCOA3, PROX2, IL16, PPHLN1, SIRT7, BAZ1A, PPRC1, CBFA2T3, ZHX1, SIRT2, MN1, TOX3, ZEB2, MED25, RARA, NCOA7, PPARA, PPARD, ELOF1, PRIM1, RELA, TCEAL7, PURA, SERTAD3, BCLAF1, SERTAD1, SERTAD2, TCEAL1, LEO1, SUZ12, ZFP710, PCGF6, STAT1, PCGF2, NR1H2, YLPM1, UNCX, LRPPRC, GATAD2A, PER2, PER1, NR6A1, NFIC, TCEA1, PKN1, SUPT16, ZFP276, CRTC2, NAB2, CTNND1, HR, ETS1, LOXL2, NR3C2, FOXQ1, CCND3, KMT5A, ZFP37, ZFP39, ZFP579, TLE3, ZFP335, TLE2, ZGPAT, ZFP219, KMT5C, LMO3, ARID5A, ZBTB33, GTF2F2, POU3F3, RSBN1, HIC1, TLE6, NPAS4, HIC2, MAF, PSPC1, KAT6B, ZFP692, ZFP691, LRIF1, KHDRBS1, KHDRBS2, AKNA, ZCCHC9, CREM, FOXO6, FOXO4, NPAS1, GTF2E1, GLIS2, ATXN3, ZKSCAN8, ZKSCAN3, SAFB2, NLRP3, LPXN, E4F1, TFAP2D, ZFHX2, CBX3, SMAD9, ESRRG, POU2F2, ESR1, ZFHX4, HOPX, NFKB1, SCRT1, SMAD7, NR4A1, MLXIPL, MRGBP, NR4A3, MAFB, SP2, CNOT1, LHX2, SP4, BHLHE40, NFE2L2, MXD4, PHF19, RNF10, ZFP523,	6,6E-04
Innate immunity	CD84, DDX3X, CYBC1, CLEC4N, PIK3CD, SIGLECG, PYCARD, C4B, TRIM8, SENP7, C4A, CLEC5A, DHX15, TNFAIP8L2, JAK3, HAVCR2, ZBP1, MAP4K2, TGTP2, FCER1G, SYK, SP110, ARID5A, TICAM1, MPEG1, IL17RA, FCGR1, HCK, NAIP5, TRAF4, IRF3, NAIP6, TMEM33, PSPC1, ADGRB1, BTK, TRIM14, MATR3, IRF5, TLR7, TLR6, RNF166, TLR13, TLR2, C1QB, CX3CR1, USP14, CSF1R, C1QA, NLRP1B, CLEC4A2, NLRX1, NLRP1A, DHX9, UNC93B1, PLD4, CFP, INAVA, CLEC7A, TIFA, DHX36, G3BP2, NLRP3, CD14, SLC15A4, GSDMD, LYN, CARD9, LY86, DAB2IP, MFHAS1, STING1, TBKBP1, C1QC	7,8E-04
