# Supplementary material for: CSF1R regulates schizophrenia-related stress response and vascular association of microglia/macrophages
Source: BMC Med. 2023 Aug 4;21:286. doi: 10.1186/s12916-023-02959-8 (PMC10403881; doi:10.1186/s12916-023-02959-8)

# CSF1R Regulates Schizophrenia-related Stress Response and Vascular Association of Microglia/Macrophages

## Supplementary materials and methods

**Clinical participants**

Complete medical histories of HCs were collected, and physical examinations were conducted for all participants to identify those with chronic medical conditions. All participants provided written informed consent. The study was approved by the Institutional Ethical Committee of Beijing Huilongguan Hospital with license No. 2017-49.

**Demographic and clinical measures**

Five factors for 29 items of Childhood Trauma Questionnaire (CTQ) include: physical abuse, emotional abuse, sexual abuse, physical neglect, and emotional neglect. Each factor has five corresponding items with five possible responses, ranging from 1 (never) to 5 (always), with the total overall trauma score ranging from 25 to 125, where higher score means more severe trauma (Bernstein et al., 2003). Reliability and validity in general Chinese population and Chinese patients with mental disorders have been demonstrated (Jiang et al., 2018).

For perceived stress scale (PSS) of 14 items~~,~~ each item has five possible responses, ranging from 0 (never) to 4 (very often), where higher score indicates higher perceived stress (Cohen et al., 1983). Reliability and validity in general Chinese population and Chinese patients with mental disorders have been demonstrated (Leung et al., 2010).

**MRI data processing**

After imputing corresponding internal anatomical instructions, 70 Desikan-Killiany (DK) atlas-based cortical and subcortical regions were extracted and data were processed with FreeSurfer software (<http://surfer.nmr.mgh.harvard.edu>) (Fischl et al., 2002; Fischl, 2012) following the ENIGMA pipeline, e.g., region-by-region visual checking and removal of incorrect values for brain segmentations (<http://enigma.usc.edu/protocols/imaging-protocols>). Bi-hemispheric regional areas and thicknesses were measured, regional and intracranial volumes (mm^3^) were calculated, and no data were excluded.

**Human and mouse RNA sample preparation**

Total RNAs were extracted using Mag-MAX^TM^ for Stabilized Blood Tubes RNA Isolation Kit (Applied Biosystems) following the manufacturer’s instructions. Mice were euthanized with CO_2_ and the prefrontal cortices were dissected and immediately stored at -80°C. Total RNAs were extracted using Trizol (Molecular Research Center). RNAs were quantified and assessed for purity by optical density ratios of 260nm/280nm and 260nm/230nm using NanoDrop spectrophotometry (ThermoFisher). RNA samples (1µg per sample) were immediately sent to the Beijing Genomics institution (BGI) and quality control on RNA samples (RIN/RQN≥7.0, 28S/18S≥1.0) was confirmed by BGI.

**GeneWeaver brain functional genomic data**

GeneWeaver, a database for the integration and analysis of heterogeneous functional genomics data (<https://geneweaver.org/>), was explored to dig out CSF1R-associated genesets annotated to contribute to human brain development. Annotated gene series GS393224 (Abnormality of brain morphology), GS393415 (Hydrocephalus), GS393709 (Abnormality of neuronal migration), GS395524 (Ventriculomegaly) were retrieved for further comparisons to overlapping blood RNA-seq DEGs and GO-BP analysis as described above.

**Mouse gene real time quantitative PCR**

Mouse total RNAs were reversely transcribed with a RevertAid First Strand cDNA Synthesis Kit (Thermo Scientific). RT-QPCR was performed by using corresponding primers and 5x HOT FIREPol® EvaGreen® qPCR Supermix (Solis BioDyne) on a PCR instrument equipped with QuantStudio 12KFlex Software v.1.2.2 (Applied Biosystems) according to the respective manufacturers’ instructions. The primers were purchased from TAG Copenhagen A/S (**Table S1**).

**Animals**

Mice from different litters were housed in 1264C Euro standard type II cages (Tecniplast) measuring 268×215×141mm. Cages containing aspen chips and wools for bedding and nesting were replaced once a week. Each cage contained 9-10 animals based on allocation after weaning. Mice were kept under standard conditions with unlimited access to food and water on a 12/12 hours (hr) light/dark cycle (light on 7am-7pm). All animal procedures in this study were performed in accordance with the European Communities Directive with the license No. 171 (01.07.2020) issued from the Estonian National Board of Animal Experiments.

**CUS and PLX3397 treatment procedures**

For CUS procedure (Yan et al., 2021), mice were exposed to a variable sequence of 7 mild and unpredictable stressors once per day for 8 consecutive weeks (wk) including: food and water deprivation overnight, rat odor and isolation overnight, restraint in 50ml tube for 2h, wet bedding and tilted cage, stroboscopic illumination overnight, flipped light/dark exposure, and swimming at 18°C for 10min. All stressors were randomly scheduled and changed daily to sustain an unpredictable procedure.

PLX3397 was dissolved at 200mg/ml in DMSO stock solution and an aliquot was freshly diluted with corn oil by 1:6.5 before use. Drug-treated mice were daily fed with Veh (100µl 15% DMSO/85% corn oil+0.5g Nutella/mouse/day for 14 days (d)) or PLX3397 (120mg/kg bodyweight, e.g., 3mg in 100µl 15% DMSO/85% corn oil+0.5g Nutella/mouse/day for 14d) added onto polystyrene Petri dishes, which were provided to mice individually for voluntary ingestion before their returning to home cages. All mice were fed daily with an equal dose of Veh for 2d in advance to get accustomed to the novel taste. Voluntary Nutella-feeding is less stressful compared to gavage for mice (Spiller et al., 2018; Cangalaya et al., 2020).

**Brain tissue processing and immunohistochemistry**

Mice were anesthetized with intraperitoneal ketamine/xylazine and transcardially perfused with PBS and 4% paraformaldehyde (PFA). After dissection, the brains were post-fixed in 4% PFA at 4°C for 1 day (d), followed by PBS washing and 30% sucrose for cryoprotection. The brains were stored at -80°C before cryosectioning at -20°C. Coronal sections were washed in PBS for 5min x 3 times with gentle rotation, followed by 15min incubation in 0.5% triton X. After PBS washings, slices were incubated with primary and secondary antibodies.

Images were taken in 1024×768 pixels at scanning velocity of 8.0 pixel/μm with Z-stacks (step=0.5μm, depth=25μm) using an Olympus microscope. 3D-reconstruction and quantification of stained cells were done using ImageJ (n=3-mice/12-sections/40~200-cells per group). For microglial morphology, images were converted into 8-bit format and “analyze particles” was used to measure whole cell and cell soma sizes, with threshold of intensity set automatically by ImageJ for whole cell, which was increased by 20% and minimal size filter set at 2.5 pixels for cell soma.

**Flow cytometry isotype controls**

Corresponding isotype control antibodies (all BioLegend) were rat IgG2b-BV605 (#135517), IgG2b-BV421 (#400639), IgG2b-BV650 (#400651), IgG2b-APC (#400219), and IgM-PE (#401611).

## Supplementary tables S1-S5: pdf files

## Supplementary figures S1-S5 and legends


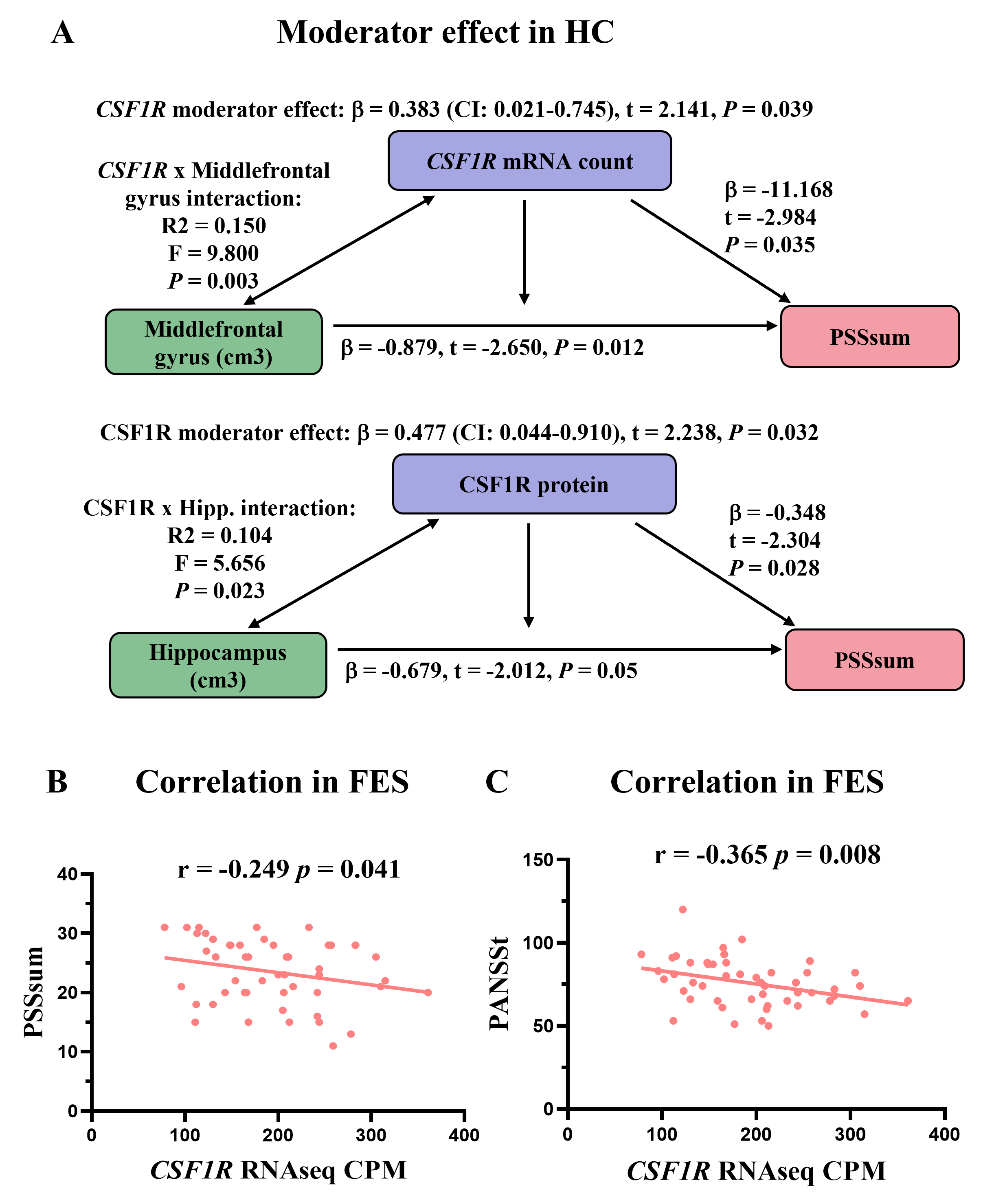


**Figure S1. CSF1R facilitated the negative associations of brain structures with PSS scores in HCs. (A)** In HCs, blood CSF1R mRNA and protein moderated the negative associations of the volumes of the middlefrontal gyrus and hippocampus (independent variables) with the PSS score (dependent variable), respectively, controlled by age, sex, and ICV (*n*=41). (**B)** In FES patients, blood *CSF1R* mRNA counts (count per million, CPM) were negatively correlated with PSS scores and (**C)** PANSSt scores (*n*=51) (Spearman’s correlation).

**Figure S2. CUS/CSF1Ri affected expression of DEGs mediating cell adhesion and Csf1r in the mouse PFC.** (**A**) RNA-seq heatmap of cell adhesion and tight junction molecules differentially expressed among 4 groups in the PFC, most of which were downregulated after CSF1Ri or CUS-CSF1Ri combination. Downregulated (in purple frame) and upregulated (in orange frame) genes are highlighted in the heatmap. The mRNA expression of (**B**) *Ang*, (**C**) *Cspg4* and (**D**) *Ptk2b*. (**E**) Csf1r mean fluorescent intensity (MFI) expressed on each hippocampal microglia (*n*=7 mice per group). CUS: chronic unpredictable stress; Ctr: Control; CSF1Ri: CSF1R inhibitor; Veh: Vehicle. **/*** *p*<0.01/0.001 compared to Ctr-Veh; Two-way ANOVA with Bonferroni’s correction.


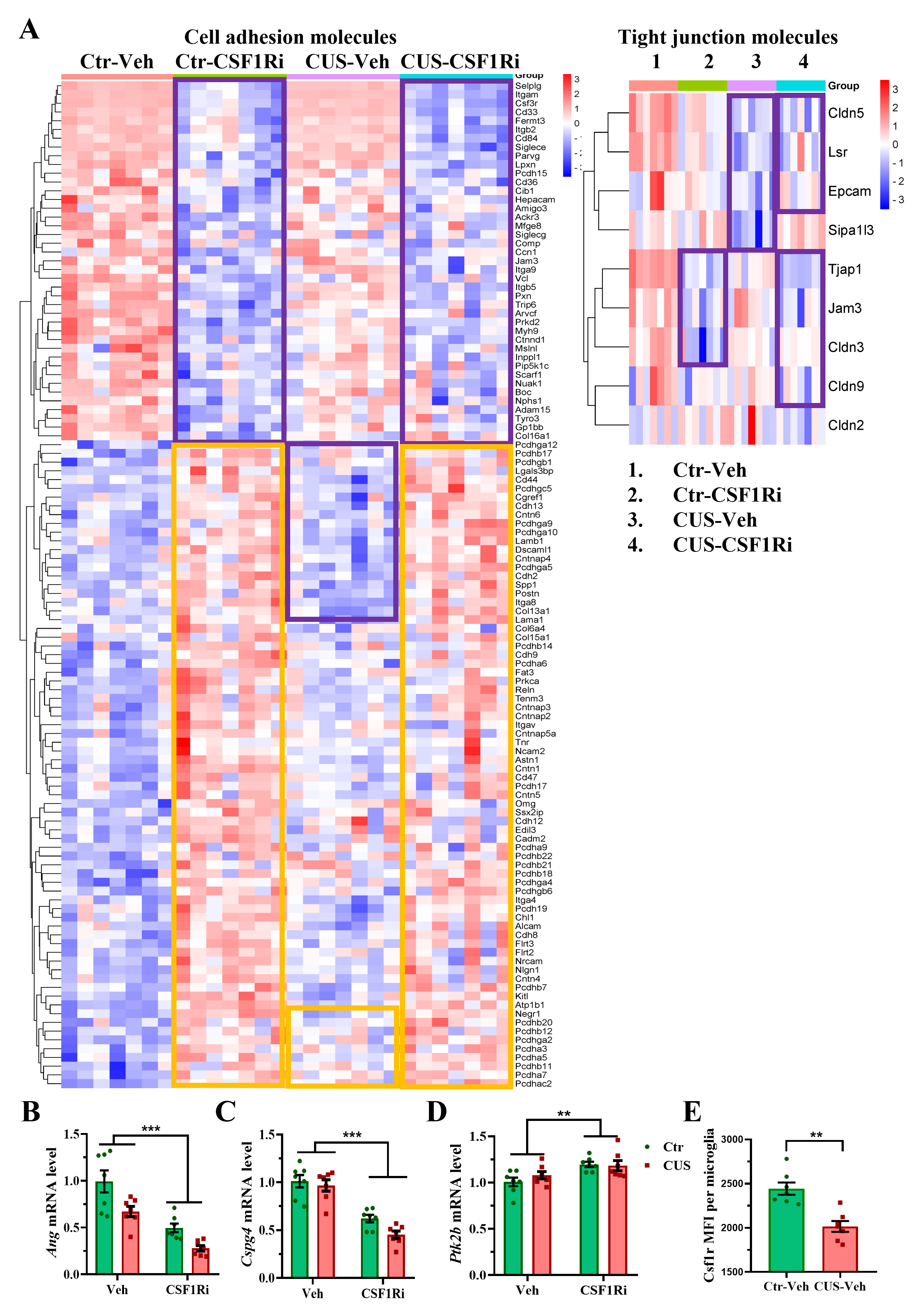

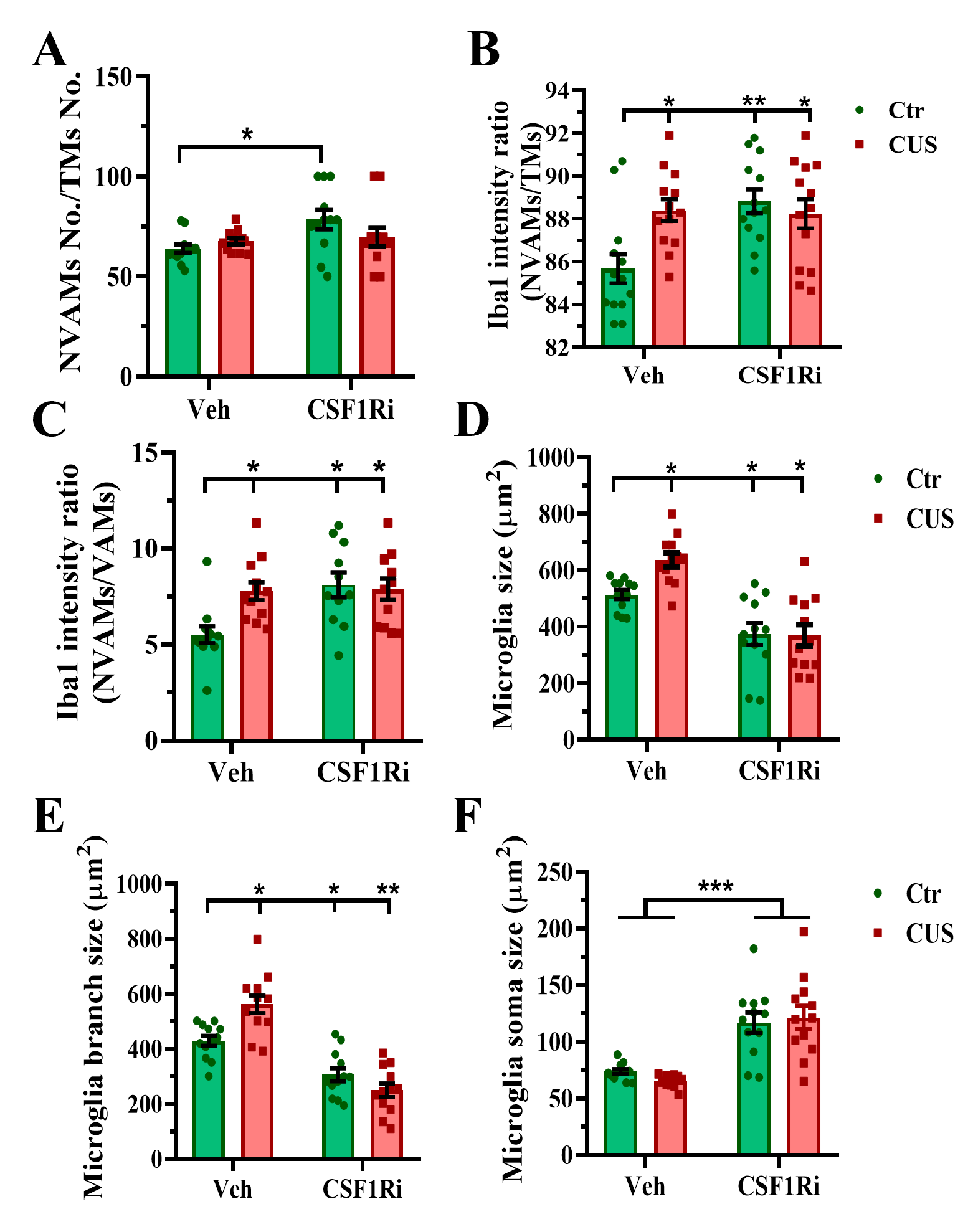


**Figure S3. Changes of IBA1 intensity and morphology in microglia/macrophages by CUS and CSF1Ri treatments in the mouse PFC.** (**A**) Relative ratio of VAMs-No./NVAMs-No., (**B**), ratio of IBA1 intensity in NVAMs versus that in TMs, and (**C**) ratio of IBA1 intensity in NVAMs versus that in VAMs. (**D**) microglia/macrophage cell, (**E**) branch sizes and (**F**) cell soma size (*n*=3 mice/12 sections/40~200 cells per group). CUS: chronic unpredictable stress; CSF1Ri: CSF1R inhibitor; Veh: vehicle; VAMs: vessel-associated microglia/macrophages; NVAMs: nonvessel-associated microglia/macrophages. Data presented as mean±SEM; */**/*** *p*<0.05/0.01/0.001 compared to Ctr-Veh. Compare group is indicated by a red vertical line. Two-way ANOVA with Bonferroni’s correction.

**Figure S4. CUS/CSF1Ri reduced CD31^+^-blood vessels and differentially affected VAMs and NVAMs in the mouse HPC.** (**A**) Representative staining of CD31 and IBA1 in the HPC (scale bar=10 µm) with enlarged VAMs and NVAMs (indicated by white and yellow arrowheads, respectively) are shown (*n*=3 mice/6 sections per group). (**B**) Blood vessel density (e.g., vessel area/total area*100%) and (**C**) total CD31 intensity. (**D**) TMs-(including VAMs and NVAMs)-No. and (**E**) total IBA1 intensity. (**F**) Ratios of VAMs-No./TMs-No. and (**G**) IBA1 intensity in VAMs/TMs. (**H**) microglia cell size, (**I**) branch sizes and (**J**) cell soma size (*n*=3 mice/6 sections/20~100 cells per group). Ctr: control; CUS: chronic unpredictable stress; CSF1Ri: CSF1R inhibitor; No.: number; TM: total microglia/macrophages; Veh: vehicle; VAMs: vessel-associated microglia/macrophages; NVAMs: nonvessel-associated microglia/macrophages. Data presented as mean±SEM; */**/*** *p*<0.05/0.01/0.001. Two-way ANOVA with Bonferroni’s correction.


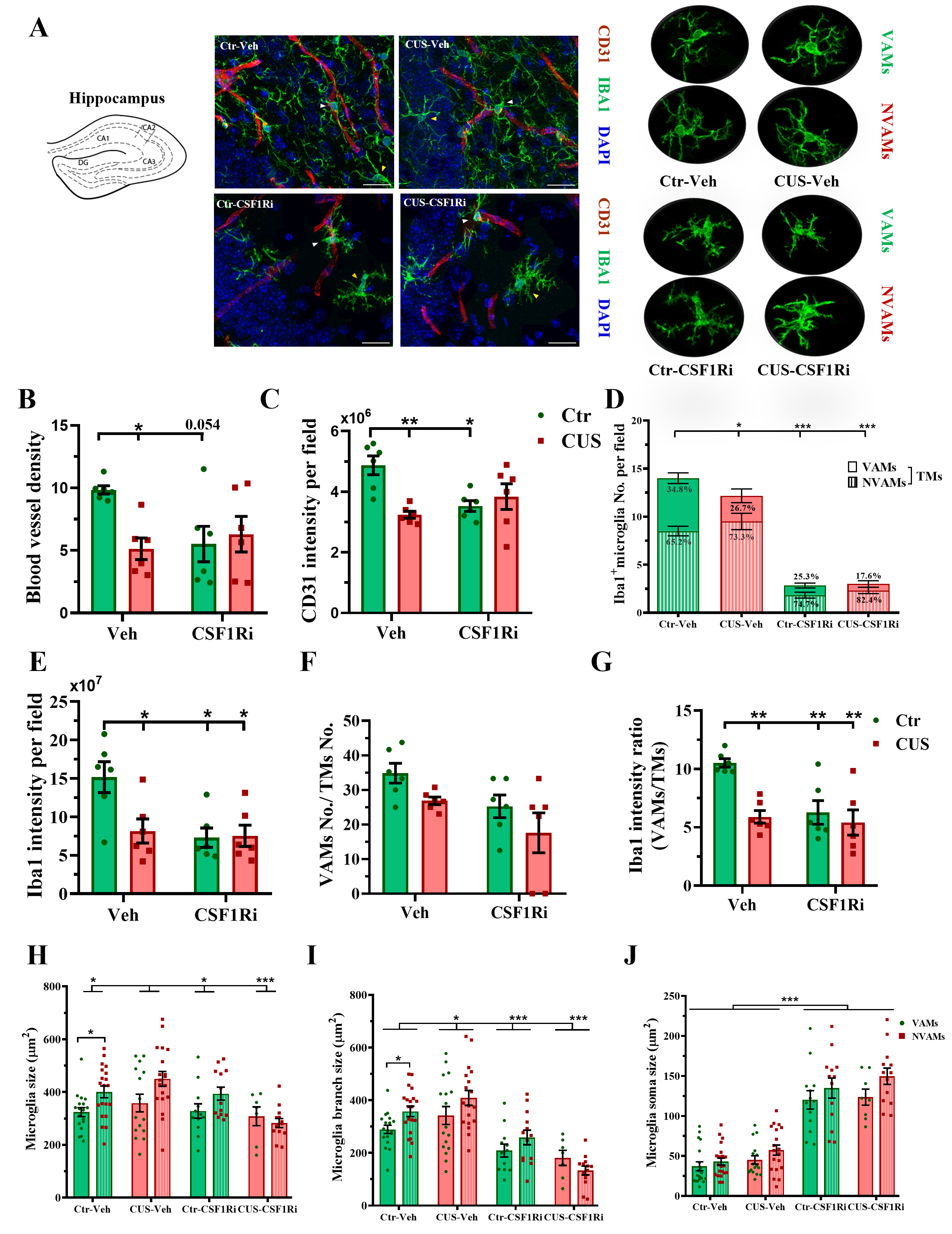


**Figure S5. Representative dot plots of negative controls used in flow cytometric analysis.** Representative plots showing negative staining by isotype control antibodies for flow markers of microglia (CD11b-iso & CD45-iso), CSF1R^+^ microglia (CSF1R-iso), OPCs (O4-iso), and astrocytes (Glast-iso) analyzed by flow cytometry.


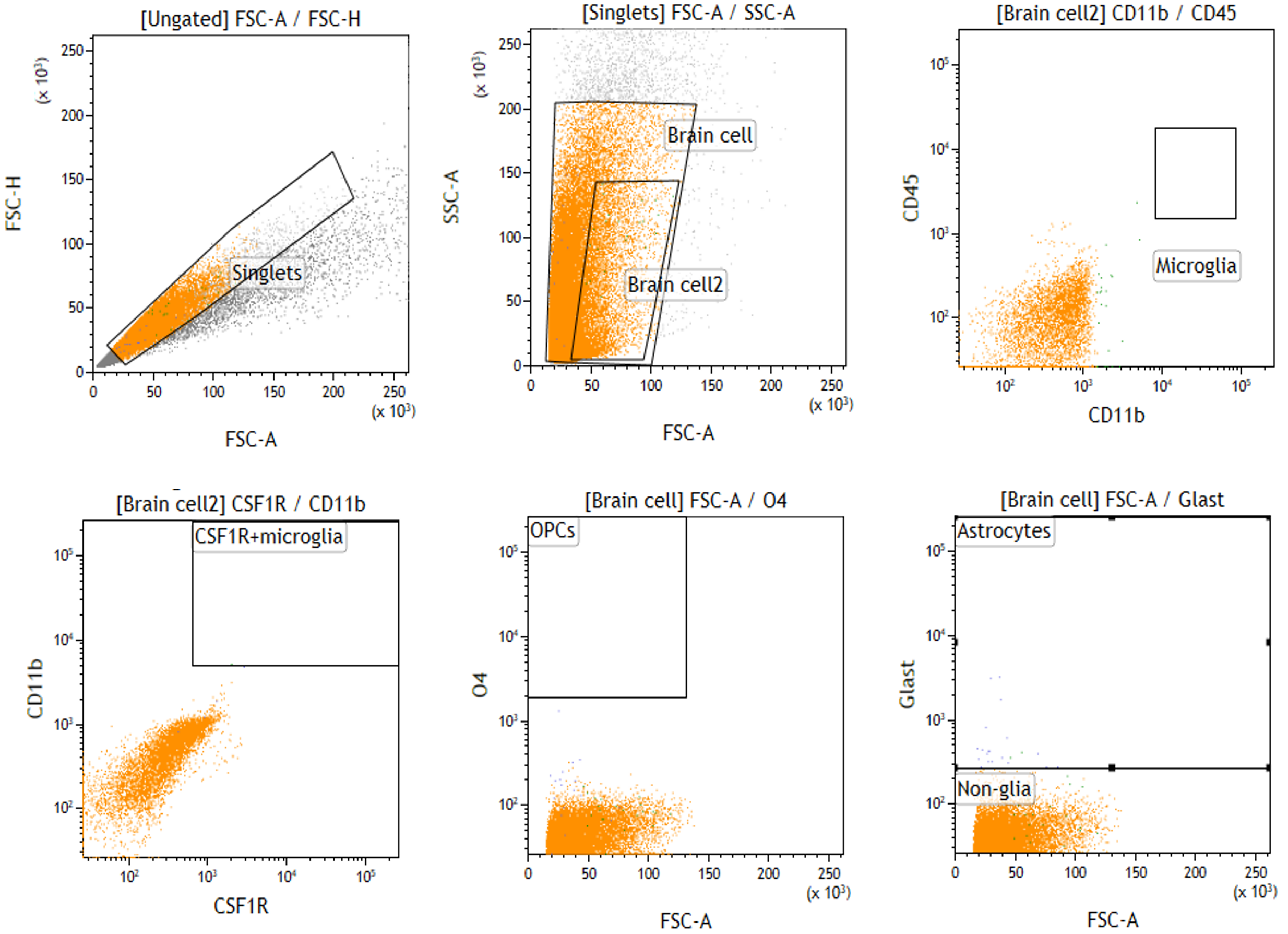

Supplement: Supplementary file 2 — Additional file 2: Fig S1. CSF1R facilitated the negative associations of brain structures with PSS scores in HCs. Fig S2. CUS/CSF1Ri affected expression of DEGs mediating cell adhesion and Csf1r in the mouse PFC. Fig S3. Changes of IBA1 intensity and morphology in microglia/macrophages by CUS and CSF1Ri treatments in the mouse PFC. Fig S4. CUS/CSF1Ri reduced CD31+-blood vessels and differentially affected VAMs and NVAMs in the mouse HPC. Fig S5. Representative dot plots of negative controls used in flow cytometric analysis. [file 12916_2023_2959_MOESM2_ESM.docx]
